# Supplementary figures and images for: Rules warp feature encoding in decision-making circuits
Source: PLoS Biol. 2020 Nov 30;18(11):e3000951. doi: 10.1371/journal.pbio.3000951 (PMC7728226; doi:10.1371/journal.pbio.3000951)

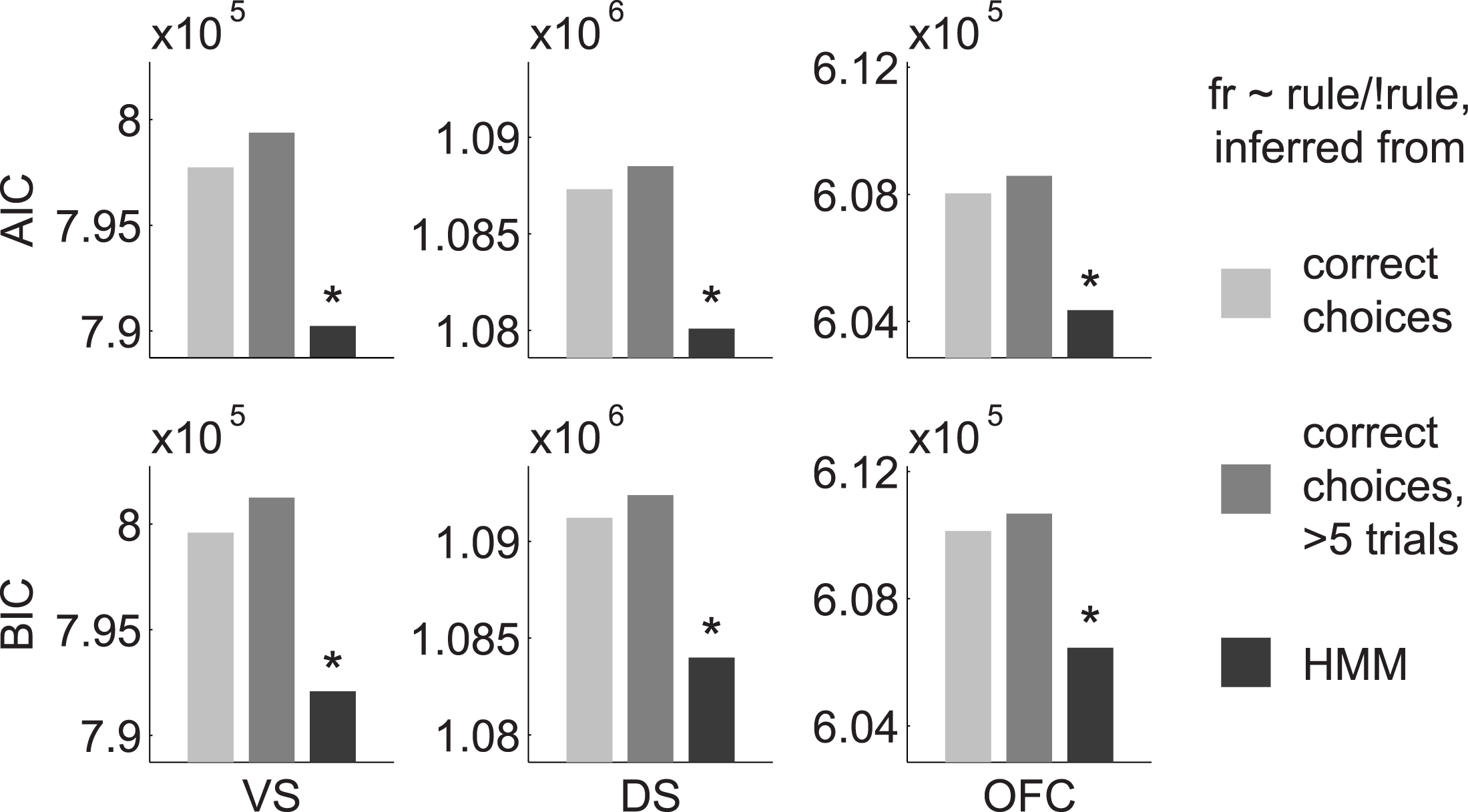

Supplement: S1 Fig — Related to Figs 1 and 2. Multiple approaches have been used to identify the rule-based decisions in tasks where the correct rule changes over blocks. The most common approach is to identify rule-based choices as the correct choices that occur within a block. Alternatively, often in tasks where rule changes are uncued, rule-based choices may be identified as the correct choices that occur after some initial burn-in or learning period (e.g., after 5 trials). Here, we instead modeled rules as the latent states underlying decisions in an HMM, then used the HMM to infer which decisions were most likely to be rule-based. To determine whether this approach was appropriate, we used model comparison to ask whether the rule labels inferred from the HMM (dark gray) better explained variance in neural activity than rule labels derived from other approaches (all correct choices within a block = light gray, all correct choices after a 5 trial burn-in period = middle gray). In model comparison, the model with the lowest AIC and BIC values is the preferred model. Within each region, AIC and BIC values were both lowest for the HMM, indicating that this approach explained the most variance in neural activity. The AIC and BIC weights for all the alternative approaches were less than 10−32, indicating very strong evidence that the HMM approach was the best. Data: https://doi.org/10.6084/m9.figshare.13139450.v1. AIC, Akaike information criterion; BIC, Bayesian information criterion; DS, dorsal striatum; HMM, hidden Markov model; OFC, orbitofrontal cortex; VS, ventral striatum. (TIF) [file pbio.3000951.s001.tif]

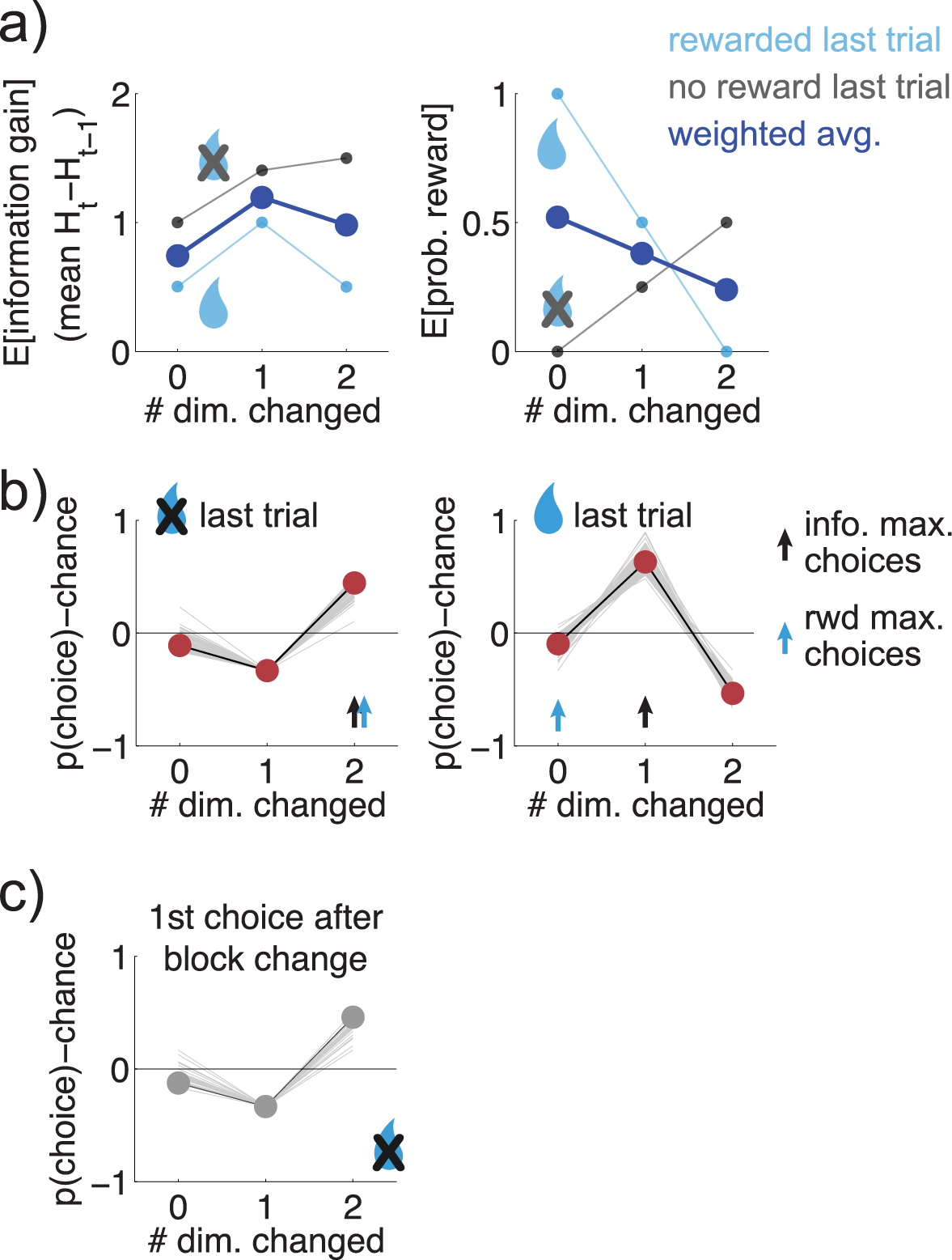

Supplement: S2 Fig — (A) The expected information gain (left) and expected reward probability (right) for choosing an option that differs in 0, 1, or 2 dimensions from the previous choice, given that the last trial was rewarded (cyan) or not rewarded (gray). The blue dotted lines shown here reflect the combination of these 2 functions, given the typical reward history on residual-state trials (i.e., a weighted average of the 2 functions, where the weight reflects the fact that 52% of residual choices follow reward delivery). (B) The choice patterns illustrated in Fig 1G, now plotted separately for residual-state choices following reward omission (left) and reward delivery (right). Conventions are the same as Fig 1G. Arrows reflect the choices that would maximize information (black arrows) or rewards (blue arrows), given that reward history, as illustrated in the previous panel. (C) Same as B, for all choices after the first omitted reward after a block change. Data: https://doi.org/10.6084/m9.figshare.13139450.v1 (TIF) [file pbio.3000951.s002.tif]

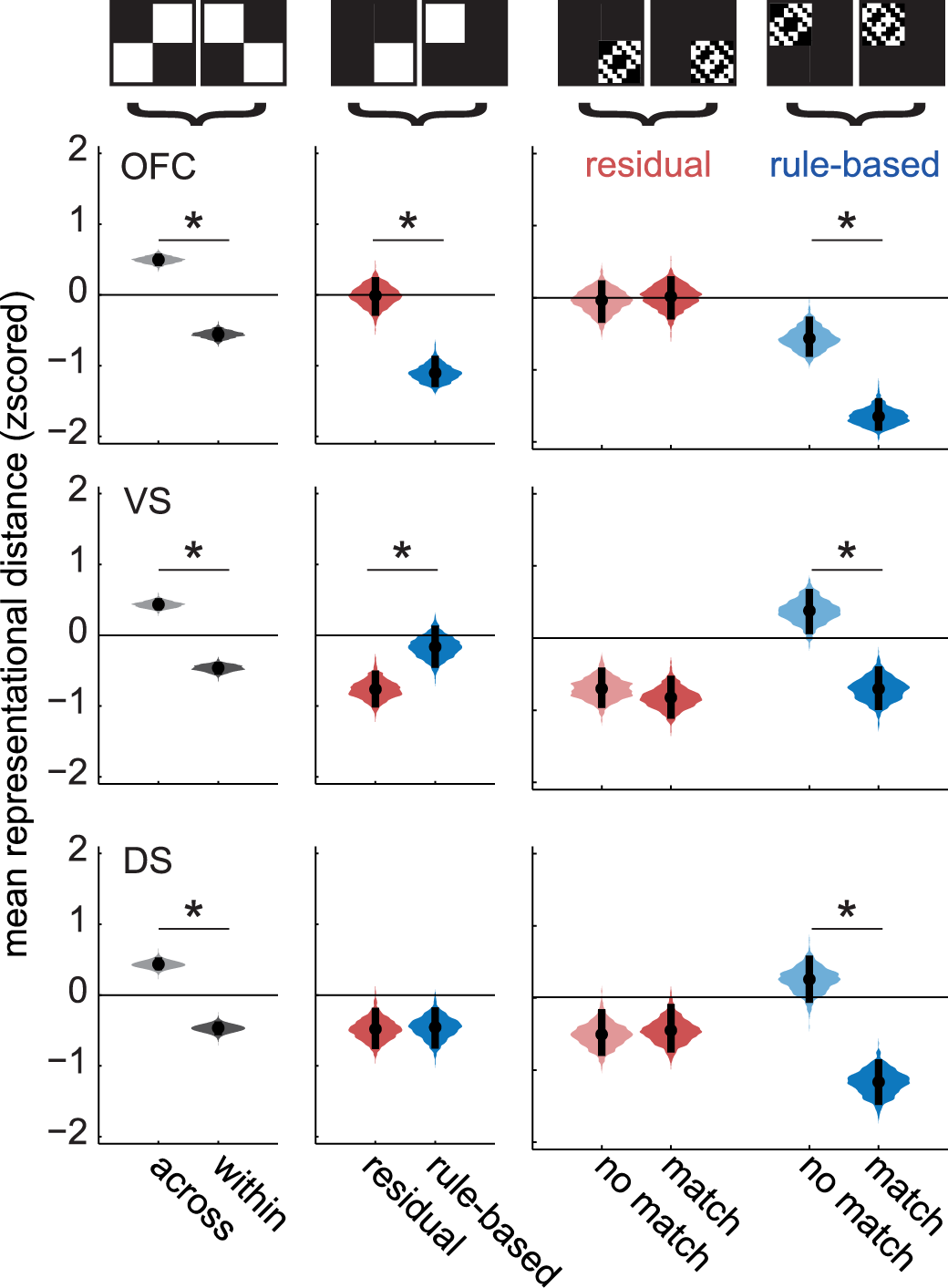

Supplement: S3 Fig — Related to Fig 5C. Distribution of means across 1,000 bootstrapped pseudopopulations for the contrasts across the representational similarity matrix. Plotted separately for OFC (top row), VS (middle), and DS (bottom). Left column) If there is a change in how choices are represented between rule-based and residual decisions, then the mean distance between rule-based and residual choice representations (light gray, average of off-diagonal blocks) should be greater than the distance within decision types (dark gray, average of on-diagonal blocks). (Middle column) If there is a change in the total representational space between decision types, then the mean distance within rule-based decisions (blue, average of top left block) should differ from residual decisions (red, average of bottom right block). (Right column) If the categorical structure of the choices is represented, then the mean distance between stimuli that share features (“match,” dark color) should be smaller than the distance between choices that do not share features (“no match,” light color). This is calculated separately for both rule-based (blue) and residual (red) decisions. Dots = mean across pseudopopulations, error bars = 95% CI, asterisks = significant contrasts, all p < 0.0001. Data: https://doi.org/10.6084/m9.figshare.13139450.v1. DS, dorsal striatum; OFC, orbitofrontal cortex; VS, ventral striatum. (TIF) [file pbio.3000951.s003.tif]

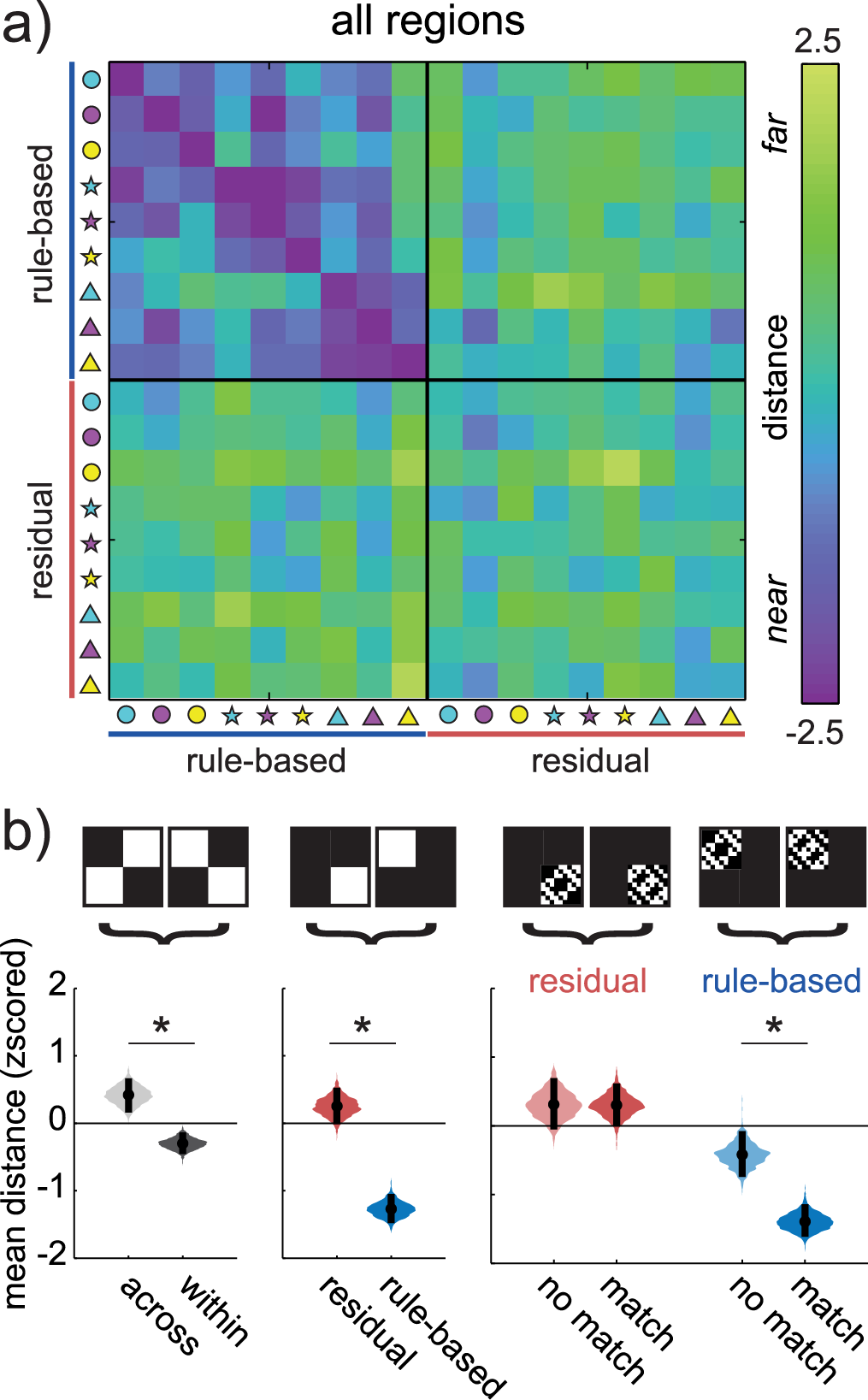

Supplement: S4 Fig — Related to Fig 5B and 5C. (A) Distances between choice representations in nonoverlapping subsets of trials. Within each cell, trials were partitioned at random into 2 equal subsets, with the constraint that each trial type was represented at least once in each subset. Then, the 2 subsets were used to construct 2 independent pseudopopulations. We then performed the same analysis as Fig 5B but calculated distances between choice representations as the distance between the representation in 1 set and the representation in the other set. After in Fig 5B, this panel illustrates 1 example partition/pseudopopulation pair. (B) The same analysis as S3 Fig, but distances are calculated from nonoverlapping subsets of the data, as in panel A. Data is combined across regions. Dots = mean across pseudopopulations, error bars = 95% CI, asterisks = significant contrasts, all p < 0.0001. Specific contrasts, from left to right. Choice representations were closer to themselves within choice type than between choice types (mean difference = 0.72, 95% CI = [0.40, 1.02], p < 0.0001, 1-sided bootstrap test). Mean representational difference was smaller during rule-based than residual choices (mean difference = 1.52, 95% CI = [1.11, 1.92], p < 0.0001). Residual choices that shared a feature were not closer together than choices that did not (mean difference = 0.01, 95% CI = [−0.31, 0.33], p = 0.48). However, rule-based choices that shared a feature were more similar than rule-based choices that did not share a feature (mean difference = 0.97, 95% CI = [0.68, 1.27]). Data: https://doi.org/10.6084/m9.figshare.13139450.v1 (TIF) [file pbio.3000951.s004.tif]

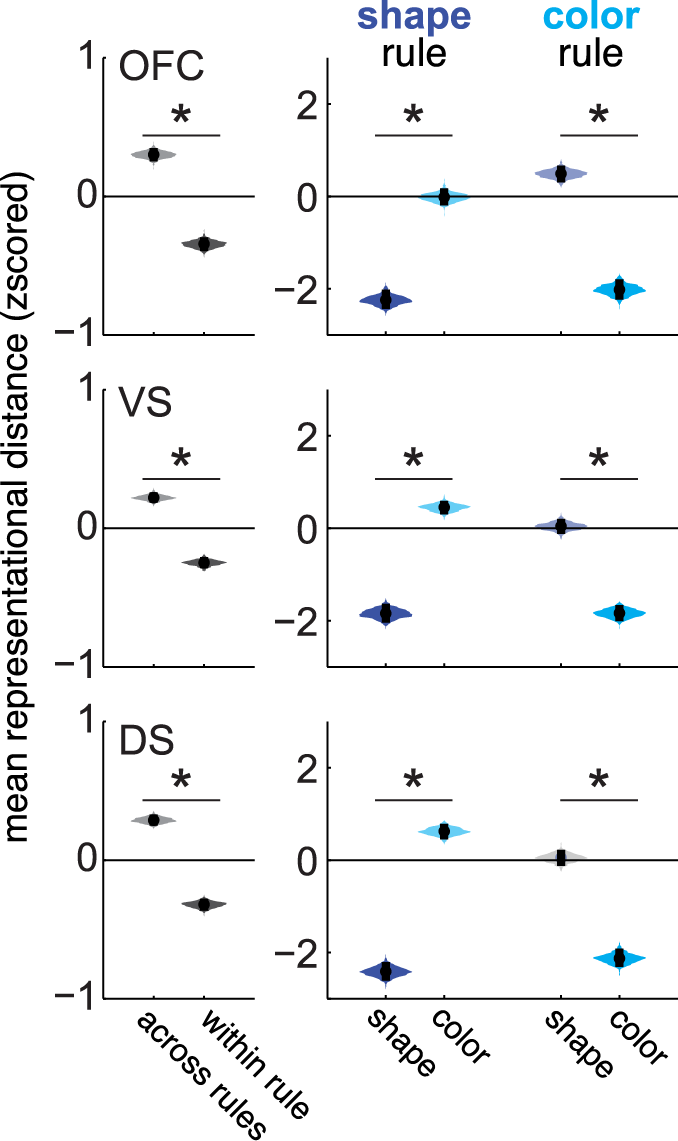

Supplement: S5 Fig — Related to Fig 6C. Distribution of means across 1,000 bootstrapped pseudopopulations for contrasts across the representational similarity matrix in Fig 6B. Plotted separately for OFC, VS, and DS (top to bottom). (Left column) A change in representation between color-rule and shape-rule decisions would increase distance between representations measured across decision types (light gray), compared to within decision types (dark gray). (Right column) Mean distance between stimuli that share the rule-relevant feature (saturated color) and rule-irrelevant feature (dim color), during shape-rule (left column) and color-rule (right column) decisions. Dots = mean, error bars = 95% CI across 1,000 bootstrapped pseudopopulations, asterisks = significant contrasts, all p < 0.001 (see Table C in S1 Text). Data: https://doi.org/10.6084/m9.figshare.13139450.v1. DS, dorsal striatum; OFC, orbitofrontal cortex; VS, ventral striatum. (TIF) [file pbio.3000951.s005.tif]

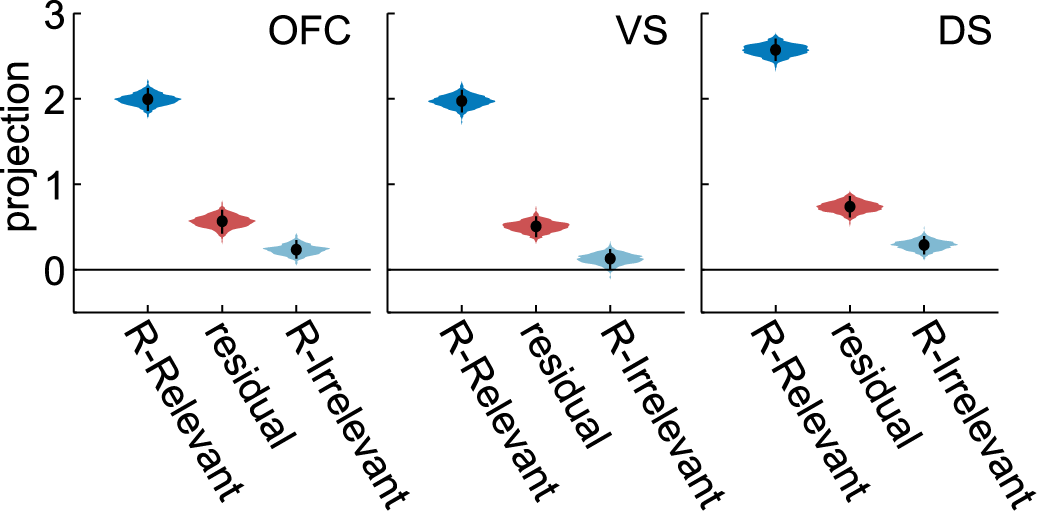

Supplement: S6 Fig — Related to Fig 7E. The projection onto the coding dimensions of the chosen features for residual decisions (red) and rule-based decisions (blue), with the latter separated according to whether the coding dimension is rule-relevant (dark blue, R-R) or rule-irrelevant (light blue, R-IR). Plotted separately for OFC, VS, and DS. Dots = mean, error bars = 95% CI across 1,000 bootstrapped pseudopopulations. Data: https://doi.org/10.6084/m9.figshare.13139450.v1. DS, dorsal striatum; OFC, orbitofrontal cortex; VS, ventral striatum. (TIF) [file pbio.3000951.s006.tif]
