# Supplementary material for: Rules warp feature encoding in decision-making circuits
Source: PLoS Biol. 2020 Nov 30;18(11):e3000951. doi: 10.1371/journal.pbio.3000951 (PMC7728226; doi:10.1371/journal.pbio.3000951)
Supplement: S1 Text — Numerical and statistical results related to Figs 5, 6 and 7. Data: https://doi.org/10.6084/m9.figshare.13139450.v1 (DOCX) [file pbio.3000951.s007.docx]

**Table A: Contrasts between rule-based and residual choices across pseudopopulations.**

|  | **OFC** | **VS** | **DS** |
| --- | --- | --- | --- |
| **Between Types**  **(across-within)** | avg. difference = 1.05  CI: [0.87, 1.24]*** | avg. difference = 0.90  CI: [0.74, 1.08]*** | avg. difference = 0.90  CI: [0.73, 1.09]*** |
| **Compression**  **(based-free)** | avg. difference = 1.09  CI: [0.62, 1.52]******* | avg. difference = -0.60  CI: [-1.11, -0.06] *** | avg. difference = -0.02  CI: [-0.57, 0.55] n.s. |
| **Rule-based**  **(shared-not)** | avg. difference = 1.08  CI: [0.86, 1.36]*** | avg. difference = 1.08  CI: [0.85, 1.32] *** | avg. difference = 1.42  CI: [1.17, 1.71] *** |
| **Residual**  **(shared-not)** | avg. difference = -0.05  CI: [-0.30, 0.18] n.s. | avg. difference = 0.12  CI: [-0.10, 0.34] n.s. | avg. difference = -0.05  CI: [-0.30, 0.20] n.s. |

Average difference between the indicated blocks of the representational similarity matrix shown in Figure 5B. CI = 95% confidence intervals across 1000 pseudopopulations. Asterisks indicate significant difference, *** p < 0.001, bootstrap test.

**Table B: Contrasts between color and shape rules within the example pseudopopulation.**

|  | **OFC** *mean ± STE* | | **VS** *mean ± STE* | | **DS** *mean ± STE* | |
| --- | --- | --- | --- | --- | --- | --- |
|  | ***Color dim.*** | ***Shape dim.*** | ***Color dim.*** | ***Shape dim.*** | ***Color dim.*** | ***Shape dim.*** |
| **Color Rule** | -0.12 ± 0.17 | -2.01 ± 0.15 | 0.41 ± 0.36 | -1.65 ± 0.27 | 0.49 ± 0.21 | -2.61 ± 0.11 |
|  | t = 8.13, [1.40, 2.38] *** | | t = 4.53, [1.10, 3.02] ** | | t = 12.88, [2.59, 3.61]*** | |
| **Shape Rule** | -1.76 ± 0.31 | 0.45 ± 0.30 | -1.92 ± 0.14 | 0.11 ± 0.14 | -2.10 ± 0.09 | 0.16 ± 0.15 |
|  | t = 5.11, [1.29, 3.12]** | | t = 19.26, [1.61, 2.45]*** | | t = 12.87, [1.88, 2.62]*** | |

Quantification of the results shown in Figure 6C. Asterisks indicate significant difference between color and shape, *** p < 0.0001, ** p < 0.001, * p < 0.01, all two-sample t-tests: t(1,16) = t-statistic, [95% CI for effect size]. All results survive correction for multiple comparisons.

**Table C: Contrasts between color and shape rules across pseudopopulations.**

|  | **OFC** | **VS** | **DS** |
| --- | --- | --- | --- |
| **Between Types**  **(across-within)** | avg. difference = 0.65  CI: [0.55, 0.74]** | avg. difference = 0.47  CI: [0.39, 0.55]** | avg. difference = 0.61  CI: [0.53, 0.69]** |
| **Shape Rule**  **(color-shape)** | avg. difference = 2.23  CI: [1.99, 2.48]****** | avg. difference = 2.30  CI: [2.10, 2.50] ** | avg. difference = 3.03  CI: [2.84, 3.23]** |
| **Color Rule**  **(shape-color)** | avg. difference = 2.51  CI: [2.30, 2.71]** | avg. difference = 1.88  CI: [1.69, 2.07] ** | avg. difference = 2.18  CI: [1.95, 2.40] ** |

Average difference between the indicated blocks of the representational similarity matrix shown in Figure 5B. CI = 95% confidence intervals across 1000 pseudopopulations. Asterisks indicate significant difference between color and shape, ** p < 0.001, bootstrap test. All results survive correction for multiple comparisons.

**Table D: Choice predictive subspace projections across pseudotrials.**

| **Condition** | **OFC** *mean (min, max)* | **VS** *mean (min, max)* | **DS** *mean (min, max)* |
| --- | --- | --- | --- |
| Unchosen feature axis | -0.89 (-0.98, -0.83) | -0.75 (-0.96, -0.49) | -1.05 (-1.17, -0.99) |
| Chosen feature axis | 0.97 (0.62, 1.32) | 0.88 (0.62, 0.97) | 1.13 (1.02, 1.23) |
| Rule-relevant (RR) | 2.11 (1.24, 2.39) | 2.00 (1.47, 2.78) | 2.47 (2.08, 3.13) |
| Residual (res.) | 0.50 (0.25, 0.72) | 0.49 (-0.04, 0.81) | 0.68 (0.53, 0.57) |
| Rule-irrelevant (IR) | 0.29 (0.03, 0.58) | 0.14 (-0.15, 0.55) | 0.22 (-0.04, 0.70) |

Quantification of the results shown in Figure 7E. All units in log odds of choice, as decoded from the example neuronal pseudopopulation in each region.

**Table E: Statistical comparisons of subspace projections across pseudotrials.**

| **Test** | **OFC** *t-stat, 95% CI* | **VS** *t-stat, 95% CI* | **DS** *t-stat, 95% CI* |
| --- | --- | --- | --- |
| Chosen ≠  Unchosen | t = 44.8, [1.78, 1.94]*** | t = 36.0, [1.53, 1.71]*** | t = 48.1, [2.09, 2.27]*** |
| RR ≠ res. | t = 21.8, [1.46, 1.75] *** | t = 20.0, [1.36, 1.65] *** | t = 23.4, [1.64, 1.94] *** |
| RR ≠ IR | t = 25.6, [1.68, 1.95] *** | t = 24.0, [1.70, 2.00] *** | t = 31.0, [2.10, 2.38] *** |
| IR ≠ res. | t = 2.9, [0.07, 0.35] ** | t = 4.49, [0.20, 0.50] *** | t = 6.2, [0.31, 0.60] *** |

Statistical tests for the results shown in Figure 7E. Asterisks indicate significant contrasts, *** p < 0.0001, ** p < 0.01, all two-sample t-tests: t(1,718) = t-statistic, [95% CI for effect size], except chosen/unchosen, where t(1,3238). All effects survive correction for multiple comparisons.

**Table F: Differences in choice predictive subspace projections across pseudopopulations.**

|  | **OFC** *mean δ, [95% CI]* | **VS** *mean δ, [95% CI]* | **DS** *mean δ, [95% CI]* |
| --- | --- | --- | --- |
| Chosen -  Unchosen | δ = 1.84, [1.76, 1.92] ** | δ = 1.63, [1.55, 1.70] ** | δ = 2.24, [2.16, 2.32] ** |
| RR – res. | δ = 1.43, [1.25, 1.61] ** | δ = 1.47, [1.28, 1.65] ** | δ = 1.84, [1.67, 2.01] ** |
| RR – IR | δ = 1.76, [1.62, 1.91] ** | δ = 1.84, [1.70, 2.00] ** | δ = 2.29, [2.13, 2.44] ** |
| res. – IR | δ = 0.33, [0.17, 0.50] ** | δ = 0.38, [0.22, 0.55] ** | δ = 0.45, [0.28, 0.61] ** |

Average of the comparisons shown in **Table S5** across 1000 bootstrapped pseudopopulations. Related to Figure 7F and Supplemental Figure 5. Asterisks indicate significant contrasts, ** p < 0.001, bootstrap test. All effects survive correction for multiple comparisons.
