# Supplementary material for: Rules warp feature encoding in decision-making circuits
Source: PLoS Biol. 2020 Nov 30;18(11):e3000951. doi: 10.1371/journal.pbio.3000951 (PMC7728226; doi:10.1371/journal.pbio.3000951)
Supplement: Attachment — Submitted filename: response2Reviewers_final_ruleEfficiencyPLOSbio.docx [file pbio.3000951.s008.docx]

**Reviewer #1:**

**To start with I would like to apologise to authors and editors that this review was submitted a couple of days over deadline, a delay caused by an unforeseen family event.**

**Ebitz, Tu & Hayden present data to test the hypothesis that the implementation of arbitrary stimulus response rules occurs through a mechanism of attentional gating through which representations within decision making circuits are modified. They use neural recordings from OFC and striatum of macaques to argue that the presence of a rule in a decision-making paradigm modified the way in which potentially to-be-chosen items were represented in these regions. They use representational similarity analysis and choice predictive subspace analysis to show that rule relevant coding dimensions dominated over rule irrelevant dimensions in pseudopopulations of recorded cells, thereby supporting a hypothesis that there is a warping of representations in decision-making circuits specifically when a rule is being applied.

There is much to like in this manuscript, it is largely well written and clear, and the analytic approach is of interest. I think there are some conceptual issues to overcome, however.**

We appreciate the reviewer’s thoughtful and detailed reading of our manuscript and kind words about the quality of our work. Below, the reviewer raised several important issues, particularly surrounding the framing of the results and the operational and conceptual definition of specific terms. These issues are thorny and the reviewer gave us much to think about as we crafted our revision. We hope that our efforts satisfactorily address these critical conceptual issues.

**Major Point

An interesting and I think useful feature of the author's approach is the use of the HMM to infer on the basis of the monkeys' responses which SR rule the monkey was currently using.**

**This permits a reasonable inference of which of the designed task rules is currently being used and is a clear improvement on using the rule in force in the task as the analysing criterion.**

The reviewer highlights one of the major contributions of this paper – the development of an improved method for inferring the rules underlying behavior. The revised manuscript further highlights this point by showing that the novel approach explains more variance in neural activity than traditional approaches (Supplemental Figure 1, included below in response to Major Point #3).

**But of course the performance rules that the HMM can infer are effectively hard coded into the model, and the contrast is a default position of "rule-free" selection. The interpretation of the neural data relies heavily on this distinction. The evidence suggests that what is going on is more complex than this, and also there are alternative interpretations for the results presented that have not been considered. My major points are therefore about the cognitive context of the study and the resulting interpretation, rather than the neural data, but I think they are fairly fundamental.**

The reviewer raises the point that there is only one type of hard-coded rule the model (e.g. the sensorimotor rules, like choose-star), and not the more complex patterns of decision-making that we observed during the “rule-free” trials (e.g. choose either a yellow option or a star option if you were rewarded for choosing yellow star on the last trial). This was an intentional choice because our goal was to identify decisions that depended on a sensorimotor rule without making assumptions about the other types of decisions that occur in this task. Modeling them as uniformly likely isn’t assuming they are random, it’s assuming we know nothing about them. However, we feel that this was insufficiently clear in the manuscript, so we have added the following language where we define the term in the results:

“In addition to 6 rule states, the model also included a “**rule-free**” state to account for all the choices that could not be explained by a simple sensorimotor rule (39). In the rule-free state, all choices were modeled as equiprobable not because they were random, but because this is the maximum entropy distribution for a categorical variable. Using the maximum entropy distribution allowed us to minimize the assumptions we had to make about these non-rule-based choices in order to model them (i.e. about what information was considered, how it was combined, how these computations changed across epochs of the task, etc.). However, it did not preclude the possibility that “rule-free” choices had, on average, some predictable fine grain structure, which indeed they did.”

Our efforts to identify the computations involved in rule-free trials appeared in Figure 1E-G and we have added new Supplemental Figure 2 to provide additional detail. These analyses show that these choices were not random, but instead complex functions of past choices and outcomes. This doesn’t mean that our model is wrong or that it would be better to try to model these complex functions directly—it simply illustrates the value and flexibility of the maximum entropy approach.

To the reviewer’s larger point, we just want to reiterate that our argument is not that “rule-free” trials are rule-free because they’re random, but because they are free of a sensorimotor rule. Because we used a maximum entropy approach to model them, they are the complete and heterogenous set of non-rule-based trials and there is no natural way to describe them as a group other than to say what they are not: based on a sensorimotor rule. However, on average, we did find that rule-free decisions were clever choices that were complex functions of the color, shape, and outcome of previous choices versus rule-based choices, which could have been made on the basis of a single if-then computation, without the need to track or consider this additional information.

**1.      The "rule-free" performance can be explained by a Win-Stay Lose-Shift rule, and so is not "rule free". Something is driving performance on "rule-free" trials, as despite being "rule-free" they are nevertheless significantly correct (1e), and in many cases this is because monkeys received trial feedback that the rule in force had changed, and then switched to a novel response. Indeed fig 1f clearly shows that this is the pattern for all "rule-free" choices. This is described in detail lines 151-162. This behaviour would appear, however, to be anything but "rule-free" - rather it adheres to the well-studied concept of "win-stay, lose-shift" rules, specifically in this case and exploratory Lose-Shift response.**

The reviewer argues that the structure of “rule-free” choices suggest that they are not actually “rule-free”, but we believe the reviewer is using a different definition of “rule-free” than the one we use in the manuscript. As described above, we have made efforts to clarify the fact that “rule-free” means only that they cannot be described with a simple sensorimotor rule, not that they are random or unstructured choices.

**WSLS is well established in the monkey behaviour literature since Harlow argued that it drives classical learning set (see also Murray & Gaffan 2006 J Exp Psychol Anim Behav Process). It is also strongly established in general cognition and AI contexts (e.g. Nowak, M., & Sigmund, K. (1993). Nature, 364(6432) ). Although WSLS is not an SR rule like those coded into the HMM, it is a performance rule, and it would appear to be driving behaviour on the trials in question. Whilst there are alternative explanations in terms of performance rules, my general point is that it makes no sense to refer to these trials as "rule-free".**

**The monkeys in this study have a vast cognitive training history (to quote from lines 637-9, "two types of foraging tasks, intertemporal choice tasks, two types of gambling tasks, attentional tasks, and a basic form of a reward-based decision task"). Many (admittedly not all) of these tasks benefit from a WSLS strategy. Indeed in many contexts WSLS is a "simpler" fall-back rule for monkeys, either when they are unable to capture a more complicated rule-set, or when reward rates mean that acquiring more complex strategies is not worth the cost.
So maybe the authors need to consider more what is driving behaviour in these "rule-free" trials, and/or incorporate something like WSLS into their behavioural / model analysis.**

We agree that there is considerable evidence that WSLS is a common and effective strategy in both humans and non-human animals and that we should consider the possibility that it was used here. Because choices were multidimensional and the monkeys used a more complex strategy, a simple WSLS strategy isn’t sufficient to explain our data, but we have added detail of the behavior to help future readers understand these choices better. To re-iterate our earlier point, these results do not imply that these choices were not “rule-free” because by “rule-free” we mean only that they were not well described by a sensorimotor rule, not that they were not clever choices that maximize reward and/or information.

Specifically, we have revised the manuscript to highlight how the pattern of switching/staying in rule-free decisions changes following reward delivery and omission. However, because the task is multidimensional, many decisions were simultaneously switch decisions in one dimension and stay decisions in another. This meant that behavior in rule-free trials was more complex than a simple WSLS strategy, but we do like the idea that it could reflect some higher order extension of a win-stay lose shift strategy—for example, it could be a win-stay lose-shift strategy in hypothesis space, rather than the traditional action space. We raise this in the discussion as follows:

“Many, but not all of the rule-free choices resembled a kind of exploratory hypothesis testing process, where a noisy win-stay lose-shift strategy was used to cycle through possible rules until the correct one was found (74-76).”

In the original manuscript, we had omitted a full discussion of the complex pattern of switch/stay preferences during rule-free trials because the major focus of the manuscript is on understanding rule-based decisions. However, we appreciate that our previous approach oversimplified the behavior and lacked clarity. Therefore, we have added Supplemental Figure 2, whose relevant panels illustrate the complete pattern of preference for switching 0, 1, or 2 choice dimensions following wins and losses, and provides benchmarks in terms of the information and reward that can be gained from using these different strategies:

**Supplemental Figure 2: Rule-free choice patterns balance reward and information maximization.** A) The expected information gain (left) and expected reward probability (right) for choosing an option that differs in 0, 1, or 2 dimensions from the previous choice, given that the last trial was rewarded (cyan) or not rewarded (gray). The blue dotted lines shown here reflect the combination of these two functions, given the typical reward history on rule-free trials (i.e. a weighted average of the two functions, reflecting the fact that 52% of rule-free trials follow reward delivery). B) The choice patterns illustrated in Figure 1G, now plotted separately for trials following reward omission (left) and reward delivery (right). Conventions are the same as Figure 1G. Arrows reflect the choices that would maximize information (black arrows) or rewards (blue arrows), given that choice history, as illustrated in the previous panel.

The reviewer points out that Figure 1F suggests strong evidence of lose-shift behavior, but this also happens to be the optimal choice in this task, as we now show in Supplemental Figure 2. Switching 2 dimensions after a loss maximizes both information and reward. Of course, this was true on average, and subjects did stay in one or more dimensions even following a loss. This could suggest a probabilistic lose-shift strategy, or it could suggest that other deterministic computations are going on, like meta reasoning about the task, reasoning about events from more than one trial back, etc. Further, on the win-stay side of the equation, behavior clearly deviates from a simple WSLS strategy. In this task, changing 1 dimension (making a decision that stays in one dimension, but switches in the other) maximizes information compared to deciding to stay in both dimensions, and these switch-1 choices are over represented following wins. This suggests an information-maximizing strategy, rather than a simple win-stay policy.

The reviewer also argues that WSLS is a rule, even if it is not a stimulus-response (SR) rule, like the color and shape rules we were focused on here. The question of what is and is not a rule is a very deep question that we struggled with in thinking about the terminology to use in this manuscript. Although the monkey’s rule-free choices were considerably more complex than WSLS choices, they could no doubt still be described as noisily following some finite (though perhaps large) set guiding principles. Does this mean that they are following a very complicated rule or should we say that they are using a strategy? We would to err on the side of classifying WSLS as a strategy, rather than as a rule, especially in the context of this manuscript, where we are using the term “rule” specifically to refer to stimulus-response rules. However, this remains a tough question and we recognize that the onus is on the manuscript to be crystal clear about these definitional issues. As such, we have replaced the use of the generic term “rules” with “stimulus-response rules” in several places in the introduction, and added the following language to the introduction:

“Many decisions require us to solve very high-dimensional problems, where the correct response is some complex function of our past choices, reward history, and the various features of the options available to us in the moment (11). However, computation takes time and energy (12), especially when performed with metabolically expensive spikes (13, 14). Thus, in practice, we use various strategies to simplify decision-making (15–17). For example, we may execute a win-stay-lose shift strategy where we need only keep track of the last choice and its outcome, then compare new options against the last choice according to the rule determined by the last outcome. There is no need calculate the value of each alternative or to represent information acquired further back into the past. Stimulus-response rules could simplify decision-making even more. To decide based on a well-learned stimulus-response rule, one need only determine whether or not an option satisfies the rule, which could be as simple as picking a berry that has the right color. This eliminates the need to represent or perform computations on any rule-irrelevant information and could thereby reduce the energetic and metabolic costs of decision-making (11, 15, 16, 18, 19).”

One might argue then that we should change “rule-free” to be “strategic” or “exploratory” or to base our terminology on some other positive definition of these choices—that we should use a definition that states what these choices are, rather than what they are not. Again, we must point out that these analyses are true *on average* of this heterogenous set of choices. Many researchers would take issue with the idea that a spontaneous failure of rule adherence when the environment has not changed is in any way strategic or exploratory. Because the group of rule-free choices includes choices like this, we feel that the most appropriate choice is to simply call them rule-free.

**2.      An explore-exploit trade-off also explains the neural data**

**There is another way of looking at these data in terms of an explore-exploit trade-off - what the HMM seems to be distinguishing is trials where the monkey is exploiting (an SR rule), vs trials which fig 1f clearly suggests are exploratory, but termed "rule-free".**

**Explore-exploit switches are known to lead to a state modulation of frontal activity in monkeys like that demonstrated in figure 2, indeed the first and senior authors have previously shown this in other cortical areas (e.g. Ebitz et al 2018 Neuron; Pearson et al 2009 Curr Biol, see also Quilodran et al 2008 Neuron). There are fewer data from the regions recorded here, but cf Costa & Averbeck 2020 J Neurosci.**

**These are established findings in the literature, and I think they provide a reasonable explanation for the initial findings, for example fig 2. So how that viewpoint interacts with the very rule-centred interpretation of the current data is critical. This (and indeed the section above) would also call into question the central claim that rule-based decisions make more efficient use of neural resources.**

The reviewer highlights a critical point: “rule-free” choices were smart choices that maximized information, so it would seem natural to describe them as exploratory choices. We are obviously very sympathetic to this perspective, which resonates with our prior work and we have added discussion of the papers cited by the reviewer to our discussion.

However, we used the term “rule-free” instead of “exploratory” because this is a heterogenous set of choices, not all of which would be unambiguously labeled as exploratory. In other words, the term “rule-free” is deliberately chosen to be a broader category and thus a more conservative term that makes fewer assumptions about the function of the choice. We have amended the text to highlight this potential link more clearly and explain why we are using the term “rule-free” instead of “exploratory”:

“Rule-free choices occurred more often, but not exclusively, at block changes (**Figure 1D**). On average, across sessions, 45% of rule-free choices (± 21% STD, 95% CI across sessions: 12 to 81%) were within 5 trials of a block change, significantly greater than the expected frequency of 21% (± 4%, t(1,127) = 14.8, p < 0.0001, paired t-test). Conversely, 35% of rule-free choices were during stable periods (10 or more trials from a change point, ± 21% STD, 95% CI across sessions: 5 to 76%), significantly less than the expected frequency of 57% (± 8%, t(1,127) = -15.9, p < 0.0001, paired t-test). This suggests that rule-free choices increased in frequency when the subjects needed to explore in order to determine what rule to follow. However, non-rule-based choices also occurred at times when monkeys could have been following rules but were not: 19.5% of the correct choices that occurred during stable periods (>5 trials after block changes) were not rule-based choices per the HMM (± 18% STD, 95% CI across sessions: 1% to 62%). We have previously argued that at least some of these choices are due to a tonic exploratory mechanism that causes us to explore both when it is valuable to do so and when it is not (39), but there is controversy about whether choices can be considered exploratory if they occur when exploration has no value (41, 42). Therefore, we will use the term “rule-free” to refer to the entire set of choices that could not be described with a sensory-response rule.”

Because we agree with the reviewer that a substantial fraction of the rule-free choices are likely exploratory, we also agree that it is important to consider the possibility that the neural effects were due to the biased sample of rule-free choices. The following paragraph now appears in the discussion, and includes our discussion of the papers the reviewer cited:

“These results are broadly consistent with theoretical work on rule-based decision-making, but the class of rule-free decisions we compared against was biased. Many, but not all of the rule-free choices resembled a kind of exploratory hypothesis testing process, where a noisy win-stay lose-shift strategy was used to cycle through possible rules until the correct one was found (74–76). In this context, it seems reasonable to interpret these results as evidence that exploration decreases the efficiency of decision-making and warps choice representations to preserve information about choice identity. This could be important for learning if it facilitates later credit assignment through some as-yet-undiscovered mechanism. However, it is not clear that we would have expected these neural results from what we know about exploration. It is true that some previous studies have reported higher net activity during exploration compared to exploitation (77, 78), but these studies conflated exploration with errors of task performance. Other studies, using different methods, do not report a net change in neural activity with exploration in decision-making regions, much less the protracted effects we show here (79–82). An explore/exploit account also cannot explain why following a specific rule would enhance the representation of rule-relevant features at the expense of irrelevant ones. Nevertheless, future work is needed to whether these results are a feature of rule-based decision-making or, alternatively, are only apparent in contrast with exploratory decisions.”

**3.      Terminology
There must be a difference between a state of responding on the basis of an inferred rule as decided by an HMM, a state of responding on the basis of an instructed/cued rule, and a state of responding based on a default acquired performance rule (c.f. learning set). The study as currently couched only draws on the first of these, yet throughout general conclusions are drawn about rule use. Some terminological clarification about the use here of the term "rule" is warranted.**

The reviewer points out that there are two major operational definitions of the term “rule” in the literature on rule-based decision-making and that it is not entirely clear how our HMM fits in to these definitions. The first one cited here is correct execution of an instructed/cued rule and the second is the execution of an acquired performance rule (what we term here the “correct features”). We agree that future work is needed to determine whether instructed rules have the same effects on neural activity as the learned rules we look at here and have amended the discussion to make this critical point:

“It will also be critical to determine whether instructed or cued rules have similar effects on neural activity, or if the representational changes we observe here are a special feature of rules acquired through experience.”

To the second point, we believe that the HMM method we present here is an improved way to operationally define “decisions that depend on acquired performance rules”, rather than a new definition of a rule. However, we recognize 1) that this was not clear in the original manuscript, and 2) we provided no comparison of our method with the traditional method. We have rectified these issues as follows. In the introduction:

“To infer what rule, if any, subjects were applying on each trial, we used a hidden Markov model (HMM; see Methods; Figure 1C). Previous rule-based sequential decision-making studies have operationally defined rule-based choices as the set of correct choices within each correct feature block. However, in this task, this approach would 1) include some unambiguously non-rule-based choices (i.e. correct responses made while guessing), and 2) exclude some unambiguously rule-based choices (i.e. perseverative rule-based decisions after the end of the block). We developed an HMM to resolves these issues, which ultimately produced trial labels that better explained variance in neural activity than these traditional approaches (Supplemental Figure 1).”

Supplemental Figure 1 now shows a direct comparison between the HMM and two more traditional approaches to separating rule-based decisions from other decisions. These new results make it clear that our approach is a better way to operationally define rule-based decisions than was used in previous studies.

***Supplemental Figure 1: The HMM explains neural activity better than other approaches.*** *Related to Figure 1 and 2. Multiple approaches have been used to identify the rule-based decisions in tasks where the correct rule changes over blocks. The most common approach is to identify rule-based choices as the correct choices that occur within a block. Alternatively, and particularly in tasks where rule changes are uncued, rule-based choices may be identified as the correct choices that occur after some initial burn-in or learning period (i.e. after 5 trials). Here, we instead modeled rules as the latent states underlying decisions in an HMM, then used the HMM to infer which decisions were most likely to be rule-based. To determine whether this approach was appropriate, we used model comparison to ask whether the rule labels inferred from the HMM (dark gray) better explained variance in neural activity than rule labels derived from more traditional approaches (all correct choices within a block = light gray, all correct choices after a 5 trial burn-in period = middle gray). In model comparison, the model with the lowest AIC and BIC values is the preferred model. Within each region, AIC and BIC values were both lowest for the HMM, indicating that this approach explained the most variance in neural activity. The AIC and BIC weights for all the alternative approaches were less than 10^-32^, indicating very strong evidence that the HMM approach was the best.*

Detail of this new analysis now appears in the methods:

***“Model comparison***. We used model comparison to determine whether the state labels inferred from the HMM better explained neural activity better than other approaches we could have used. Firing rates of each neuron *i* were modeled as Poisson distributed, with the mean rate dependent on whether or not each trial *t* was rule-based:

The logical “rule” was defined in one of three ways: 1) rule versus not rule as inferred from the HMM, 2) correct versus incorrect choices, 3) correct responses 5 or more trials after the block change versus other choices. We then calculated the log likelihood of the three models and, from that, their Akaike information criterion (AIC) and Bayesian information criterion (BIC) values (Matlab: aicbic.m). AIC and BIC provide measures of the relative likelihood of sets of models, but their units are arbitrary, so they are transformed into relative likelihoods (AIC or BIC weights) for quantitative comparison.”

**On a more general cognitive level, I would suggest that characterising behaviour as simply "rule-free", especially when performance is adaptive or high, doesn't make an awful lot of sense. The animals are making directed responses, this behaviour is thus driven by something. I accept that it's not being driven by a specific SR rule imposed in the task, but monkeys (and humans) don't simply respond using the rules we wanted to impose.**

This returns to the issue of understanding that the term “rule-free” does not mean or imply that choices were random or unstructured. We have addressed this point piecemeal in response to previous questions, but to reiterate, we have changed manuscript to clarify the definition of rule-free:

“In addition to 6 rule states, the model also included a “**rule-free**” state to account for all the choices that could not be explained by a simple sensorimotor rule (39). In the rule-free state, all choices were modeled as equiprobable not because they were random, but because this is the maximum entropy distribution for a categorical variable. Using the maximum entropy distribution allowed us to minimize the assumptions we had to make about these non-rule-based choices in order to model them (i.e. about what information was considered, how it was combined, how these computations changed across epochs of the task, etc.). However, it did not preclude the possibility that “rule-free” choices had, on average, some predictable fine grain structure, which indeed they did.”

In terms of understanding what is going on during rule-free trials, we are very sympathetic to the reviewer’s point that monkeys and humans do all kinds of things beyond what the task imposes. (Indeed, this was our entire motivation in using the HMM at all, not to mention modeling rule-free choices as equiprobable so as to not make assumptions before analyzing them!) We have done our best to detail what is going on during these trials via 4 lengthy paragraphs in the results (p 3-5) and multiple figure panels (Figure 1E-G, Supplemental Figure 2). However, it is important to note that these trials were heterogenous—they are the complete set of non-rule-labeled trials—which makes it difficult to ascribe a specific high-level cognitive label to them as a group. We can say on average that these choices were clever, information-seeking choices, but some of these choices also occurred when information seeking had no value, suggesting that multiple different mechanisms were probably driving this class of trials. Ultimately, labeling these trials in terms of what they lacked (“rule-free”) was the only succinct and accurate way we could describe them.

**So in summary I think the authors must consider these points in the context of what is nevertheless interesting data and a useful and well established task. I would actually argue that there is something much more interesting going on than the very simple story the authors present, related to context-driven switches between types of rule. But of course my role here is not tell the authors about the study I would have done with their nice data.**

We appreciate the reviewer’s thoughtful feedback and we did indeed have much to think about (and many ideas for future directions!) in responding to these comments. Thank you.

**Other points to consider

A number of comments are made throughout about reducing the energetic costs through the use of rules, though a link to the data is not really made explicit. I would imagine that the authors are referring to the reduced overall firing rate, but at the same time the encoding of rule-relevant dimensions seems to increase more than the compression of irrelevant dimensions. Is the argument here that the first effect has energetic consequences and not the second? If so, why?**

We apologize for the confusion here, re-reading the manuscript now, it seems we only used the term “energetic costs” in the abstract and discussion, but did not link it to the specific analyses that grounded this claim in the results. First, we have foreshadowed this result in new language in the introduction:

“However, computation takes time and energy (12), especially when performed with metabolically expensive spikes (13, 14).”

Second, we have revised the relevant section of the results to draw a clearer line between the conclusions and the data:

“Spikes are energetically costly (13, 14), so a distributed decrease in neural activity during rule-based decisions could reflect decrease in the energetic costs of rule-based decisions, compared to decisions that require navigating more complex decision-spaces. Alternatively, the decrease in spike rates could suggest that OFC, VS, and DS are just disengaged during rule-based decisions—perhaps they fire fewer spikes because they are simply encoding less information, for example. Ultimately, a more efficient code requires both 1) that fewer spikes be expended (as we have seen), and 2) that information not be lost, despite this decrease in firing rate. Therefore, we next asked whether neurons in these regions encoded choice information and whether the energetic (spiking) cost of encoding this information was altered during rule-based decisions.”

To the reviewer’s second point, our sense is that spikes are our best measure of the metabolic costs of computation. So, if more of information is encoded in fewer spikes, we would take that as evidence that the metabolic cost of encoding that information are reduced. Of course, we need to be clear about this assumption. We have added the following language to the brief summary of theses analyses in the results:

“Second, the information per spike also increased during rule-based decisions in all three regions (VS, rule-based: 0.012 bits per spike ± 0.005, rule-free: 0.004 ± 0.001, p < 0.0002, z = 3.84, DS, rule-based: 0.008 ± 0.001, rule-free: 0.005 ± 0.0006, p < 0.0001, z = 6.11, and OFC, rule-based: 0.009 ± 0.001, rule-free: 0.007 ± 0.001, p < 0.002, z = 3.11; all effects survive correction for multiple comparisons). Because spikes are metabolically costly (13, 14), the fact that more information was conveyed per spike suggests that rule-based decisions are more energetically efficient than rule-free decisions. Fewer spikes were wasted on computations that were irrelevant to the ultimate choice. This may suggest that using a rule changes the way that neurons are tuned for choice in these regions.”

**The suggestion (lines 70-72) that refs 12 and 13 fail to find evidence for an attentional gating hypothesis is, I think, not the completely correct characterisation of these studies. But they do propose alternative mechanisms, and it would be worth the authors discussing how these mechanisms can be integrated with the mechanisms they reveal.**

We agree that our discussion of these studies was not as precise as it should have been. We have nearly completely re-written the introduction in response to some later comments from this reviewer and from the other reviewers, but the specific passages that reference these papers (now numbered 21 and 22) now read:

“One influential hypothesis is that the brain implements rules and other executive functions as a kind of attentional gate that enhances the representation of relevant features of the world at the expense of irrelevant features (20). Eliminating the early sensory encoding of irrelevant information would certainly reduce the costs of decision-making. However, tests of this hypothesis in both prefrontal cortex (21, 22) and early sensory cortex (23, 24) have failed to find evidence of early feature selection. Information about irrelevant features of behaviorally relevant stimuli is still strongly encoded in these regions, even when it can interfere with task performance (4, 21, 22).”

This paragraph focused on one null empirical result in these papers, but we also highlight the mechanism suggested by the computational model in reference 22 (Mante, Susillo, Newsome & Shenoy, 2013) in other places in the manuscript. This includes the following line in the introduction:

“Here, we build on theoretical work that suggests that executive processes like rules may alter how sensory information is transformed into motor responses (22, 35).”

There is also the following line in a new section of the results on whether these effects are a simple consequence of feature-based attention (in response to Reviewer #3):

“However, these are changes in choice representations, not stimulus representations, and, as such, could be due solely to late changes in the decision-making process itself (22, 35) rather than early changes in visual attention.”

And we have amended the discussion to add additional detail of how we see our work situated in the context of these papers:

“The idea that cognitive processes warp how the brain represents information has support in both influential theories of prefrontal function (20) and modern views at the intersection of executive function and selective visual attention (53, 58, 83–85). Our results strongly support the idea of representational warping: showing that rules not only gate which stimuli are represented in the neural code (86), but also *how* different stimuli are represented. This result is particularly significant because several previous single neuron studies did not report evidence that task demands can gate or warp the representation of low-level sensory features in the brain ((4, 21–24), though compare with fMRI results (87, 88)). Future work is necessary to determine whether the critical difference between our study and previous single unit studies is one of task design, analysis method, or the fact that this study targeted limbic regions commonly implicated in decision-making, rather than prefrontal or extrastriate regions linked to controlling or executing visual attention (21–24). Certainly, one straightforward way to produce broadly distributed representational changes would be through a change in visual attention, either at the level of the extrastriate cortex (26–28) or in the prefrontal regions that direct featural attention (89). Early feature selection could, in theory, alter the way that stimulus features are represented within every region that receives ascending visual information. We do report some evidence in favor of this idea here. However, like many attentional effects, early feature selection was modest and some kind of nonlinear amplifying process would still be needed to transform modest biases in stimulus representations into substantial biases in choice representations. Further, similar effects have not been reported in regions more clearly implicated in visual attention (4, 21–24) and it is still not entirely clear that feature-based attention *can* prioritize one feature of a stimulus at the expense of another (27–29, 32–34).

A change in sensory encoding is not the only way to produce changes in how choice information is represented in neural populations (22, 35). The effects we report here could also be due to other selective mechanisms, including changes in the intrinsic dynamics of populations of decision-making neurons (22, 35), an adjustment in neuronal gain coming from dorsal anterior cingulate (72, 90), or amplification through recurrent interactions between these regions and/or through the thalamus (91)..”

**Fig 3 and associated text lines 215-222. The concept of "automatic or deliberative decision-making regions" as driving a very specific hypothesis to be tested is unclear to me. Whilst the idea is described in the introduction (87-96), what is not clear (either from the text or from the cited references) is how one would define an entire region as involved in deliberative decision making. As posed, this distinction feels a little phrenological, and I'm not sure this is how the authors mean it. If it is to be used, I feel a strong, formal, and evidence-based definition of what makes a whole area "deliberative" is necessary, along with a justification for the classification of whole brain areas as one or the other.**

**Perhaps related, I find the "handoff" hypothesis under specified with respect to the supposedly contrasting warping hypothesis. The assertions that the evidence support warping are well justified, the parallel dismissal of handoff is much less clear - what would be the true neural correlates of this hypothesis, and do the authors really have the neural data (notably enough cortical regions) to test it? More generally, and related to the comments at the start, the idea of binary opposing hypotheses feels a little forced, when there is so much going on.**   

Our understanding from both this reviewer and from Reviewer #2 is that the automatic/deliberative “handoff hypothesis” we described was not entirely clear and did not seem to help with the understanding of the main results. Further, while our results are clearly better aligned with the warping hypothesis, we cannot definitively reject the handoff hypothesis (which could still be happening in regions we did not record from, for example). Therefore, we have decided to take the reviewers’ suggestion and refocus the paper on the warping hypothesis that motivated the vast majority of our analyses.

We have made many changes to the manuscript to address this concern, including a re-written introduction, the removal of hypotheses illustrated in Figure panel 3C, and the removal of all references to the handoff hypothesis from the text and figure legends. Citations to the papers used to motivate this hypothesis remain, but are now used to situate our results in a literature focused on the functional differences between these three regions. Our sense is that these changes did not substantially alter the presentation or interpretation of the data, suggesting that the reviewer was correct to point out that this hypothesis was clutter in an already dense paper.

**Fig 4. Is pretty confusing. It doesn't help that there is no E) on the figure but there is in the legend (it appears that the current C) should be both C) and D)), and that the text (e.g. line 390) refers to 4C in the context of "identity-category index" when these results appear to be presented in 4D. Further, the legend could be much clearer, and all this made the figure really hard to interpret. Indications of the significant effects claimed in the text would also be helpful.**

We apologize that these panels were mislabeled. We have corrected the figure legend and the reference to the identity-category index panel in the main text. We have also added indicators to identify the significant effects to this figure (and also to the next figure, which was also missing these indicators). Finally, we have amended the caption of Figure 4 (now Figure 5) to be more clear:

**Figure 5: Changes in choice representations across rule-based and rule-free decision-making.** *A) Separately recorded neurons were combined into rule-based and rule-free pseudopopulations (left). This allowed us to estimate the position of different types of pseudotrials in neuron-dimensional state space (middle). To determine how different task conditions affected the geometry of neural representations, the average distance between different types of choices (here, between cyan circle and magenta circles) is calculated and used to populate a representational similarity matrix (right). The example matrix shows the structure this matrix will have if choices that share a color or shape are represented by more similar patterns of activity across neurons than than choices that do not share features (i.e. if these choices are closer together in neuronal state space). B) The representational similarity matrix for both rule-based (upper left) and rule-free (bottom-right) decisions, calculated across all regions, as well as the similarity between rule-based and rule-free representations (off-diagonal blocks). C) Specific contrasts across the representational similarity matrix for all regions, then separately for OFC (top row), VS (middle) and DS (bottom). Left column) If there is a change in how choices are represented between rule-based and rule-free decisions, then the mean distance between rule-free and rule-based choice representations (light gray, average of off-diagonal blocks) should be greater than the distance within decision types (dark gray, average of on-diagonal blocks). Middle column) If there is a change in the total representational space between decision types, then the mean distance within rule-based decisions (blue, average of top left block) should differ from rule-free decisions (red, average of bottom right block). Right column) If the categorical structure of the choices is represented, then the mean distance between stimuli that share features (“match”, dark color) should be smaller than the distance between choices that do not share features (“no match”, light color). This is calculated separately for both rule-free (red) and rule-based (blue) decisions. Error bars = STE across unique matrix elements, asterisks = significant contrasts in both the example pseudopopulation (all p < 0.001, paired t-test) and across pseudopopulations (all p < 0.001, bootstrap test, Suppemental Figure 2). D) The identity-category index for single neurons in OFC (left), VS (middle) and DS (left). Error bars = STE across neurons, asterisks = significant contrasts.*

**Line 844-846 "All reported pseudopopulation results come from a single pseudopopulation, but were confirmed by bootstrap tests across 1000 randomly re-seeded pseudopopulations." Can we see the outcome of these bootstraps to have an idea of across pseudopopulation variation? Unless I have misunderstood something, this is not currently presented.**

We have now added detail of the additional bootstrap analyses to the main text. This includes extensive additional text, two new Supplemental tables, and three new supplemental figures (Supplemental Figure 3, 4, and 5) which recapitulate all of the major results in the paper (i.e. Figures 5C, 6C, and 7E). These new analyses show that all the major results do replicate across 1000 bootstrapped samples. However, we had not checked one minor effect—the compression of representational space in DS during rule-based decisions—and here we found that this did not replicate across 1000 samples, so we have amended the results, figure legend, and discussion accordingly.

Bootstrapped population analyses related to Figure 5C appear in Supplemental Table 1:

**Supplemental Table 1: Contrasts between rule-free and rule-based choices across pseudopopulations.**

|  | **OFC** | **VS** | **DS** |
| --- | --- | --- | --- |
| **Between Types**  **(across-within)** | avg. difference = 1.05  CI: [0.87, 1.24]*** | avg. difference = 0.90  CI: [0.74, 1.08]*** | avg. difference = 0.90  CI: [0.73, 1.09]*** |
| **Compression**  **(based-free)** | avg. difference = 1.09  CI: [0.62, 1.52]******* | avg. difference = -0.60  CI: [-1.11, -0.06] *** | avg. difference = -0.02  CI: [-0.57, 0.55] n.s. |
| **Rule-based**  **(shared-not)** | avg. difference = 1.08  CI: [0.86, 1.36]*** | avg. difference = 1.08  CI: [0.85, 1.32] *** | avg. difference = 1.42  CI: [1.17, 1.71] *** |
| **Rule-free**  **(shared-not)** | avg. difference = -0.05  CI: [-0.30, 0.18] n.s. | avg. difference = 0.12  CI: [-0.10, 0.34] n.s. | avg. difference = -0.05  CI: [-0.30, 0.20] n.s. |

Average difference between the indicated blocks of the representational similarity matrix shown in Figure 5B. CI = 95% confidence intervals across 1000 pseudopopulations. Asterisks indicate significant change from rule-free, *** p < 0.001, bootstrap test.

These results are illustrated in new Supplemental Figure 3:

**Supplemental Figure 3: Changes in choice representations across rule-based and rule-free decision-making across pseudopopulations.** *Related to Figure 5C. Distribution of means across 1000 bootstrapped pseudopopulations for the contrasts across the representational similarity matrix. Plotted separately for OFC (top row), VS (middle) and DS (bottom). Left column) If there is a change in how choices are represented between rule-based and rule-free decisions, then the mean distance between rule-free and rule-based choice representations (light gray, average of off-diagonal blocks) should be greater than the distance within decision types (dark gray, average of on-diagonal blocks). Middle column) If there is a change in the total representational space between decision types, then the mean distance within rule-based decisions (blue, average of top left block) should differ from rule-free decisions (red, average of bottom right block). Right column) If the categorical structure of the choices is represented, then the mean distance between stimuli that share features (“match”, dark color) should be smaller than the distance between choices that do not share features (“no match”, light color). This is calculated separately for both rule-free (red) and rule-based (blue) decisions. Dots = mean across pseudopopulations, error bars = 95% CI, asterisks = significant contrasts, all p < 0.001 (see Table S3).*

The pseudopopulation analyses for Figure 6 now appear in new Supplemental Table 3:

**Supplemental Table 3: Contrasts between color and shape rules across pseudopopulations.**

|  | **OFC** | **VS** | **DS** |
| --- | --- | --- | --- |
| **Between Types**  **(across-within)** | avg. difference = 0.65  CI: [0.55, 0.74]*** | avg. difference = 0.47  CI: [0.39, 0.55]*** | avg. difference = 0.61  CI: [0.53, 0.69]*** |
| **Shape Rule**  **(color-shape)** | avg. difference = 2.23  CI: [1.99, 2.48]******* | avg. difference = 2.30  CI: [2.10, 2.50] *** | avg. difference = 3.03  CI: [2.84, 3.23]*** |
| **Color Rule**  **(shape-color)** | avg. difference = 2.51  CI: [2.30, 2.71]*** | avg. difference = 1.88  CI: [1.69, 2.07] *** | avg. difference = 2.18  CI: [1.95, 2.40] *** |

Average difference between the indicated blocks of the representational similarity matrix shown in Figure 6B. CI = 95% confidence intervals across 1000 pseudopopulations. Asterisks indicate significant change from rule-free, *** p < 0.001, bootstrap test.

These results are illustrated in new Supplemental Figure 4:

**Supplemental Figure 4: Changes in choice representations during color and shape rules across pseudopopulations.** *Related to Figure 6C. Distribution of means across 1000 bootstrapped pseudopopulations for contrasts across the representational similarity matrix in Figure 6B. Plotted separately for OFC, VS, and DS (top to bottom). Left column) A change in representation between color-rule and shape-rule decisions would increase distance between representations measured across decision types (light gray), compared to within decision types (dark gray). Right column) Mean distance between stimuli that share the rule-relevant feature (saturated color) and rule-irrelevant feature (dim color), during shape-rule (left column) and color-rule (right column) decisions. Dots = mean, error bars = 95% CI across 1000.*

The pseudopopulation analyses for Figure 7 are in Supplemental Table 6:

**Supplemental Table 6: Differences in choice predictive subspace projections across pseudopopulations.**

|  | **OFC** *mean δ, [95% CI]* | **VS** *mean δ, [95% CI]* | **DS** *mean δ, [95% CI]* |
| --- | --- | --- | --- |
| Chosen -  Unchosen | δ = 1.84, [1.76, 1.92] *** | δ = 1.63, [1.55, 1.70] *** | δ = 2.24, [2.16, 2.32] *** |
| RR - FR | δ = 1.43, [1.25, 1.61] *** | δ = 1.47, [1.28, 1.65] *** | δ = 1.84, [1.67, 2.01] *** |
| RR - IR | δ = 1.76, [1.62, 1.91] *** | δ = 1.84, [1.70, 2.00] *** | δ = 2.29, [2.13, 2.44] *** |
| FR – IR | δ = 0.33, [0.17, 0.50] *** | δ = 0.38, [0.22, 0.55] *** | δ = 0.45, [0.28, 0.61] *** |

*Average of the comparisons shown in* ***Table S5*** *across 1000 bootstrapped pseudopopulations. Related to Figure 7F and Supplemental Figure 5. Asterisks indicate significant change from rule-free, *** p < 0.0001, bootstrap test.*

And these results are illustrated in Supplemental Figure 5:

**“Supplemental Figure 5: Changes in choice representations within a choice-predictive subspace, distribution of means across pseudopopulations.** Related to Figure 7E. The projection onto the coding dimensions of the chosen features for rule-free decisions (red, RF) and rule-based decisions (blue), with the latter separated according to whether the coding dimension is rule-relevant (dark blue, R-R, or rule-irrelevant (light blue, R-IR). Plotted separately for OFC, VS, and DS. Dots = mean, error bars = 95% CI across 1000 bootstrapped pseudopopulations”

**Figure comments.
1e and 1f would be clearer as boxplots or similar. Notably in 1e the comparison of red and grey is visually misleading.**

We have replaced the errorbar plots in 1E and 1F with violin plots (illustrating the distribution) in addition to errorbars that now reflect 95% CI around the mean (dot). In Figure 1E, the gray dots were the raw data used to calculate the original bar plot, so we didn’t intend a comparison across plotting types, sorry about that. We removed the raw data in favor of the violin plot which is more clear.

**S1b would also be better presented as boxplots and it would be helpful to have axes that permit direct comparison between the 3 plots…**

There are large baseline differences between regions that make it difficult to illustrate the differences between them on the same y-axis without having to increase the height of the plot substantially. However, it would be helpful to explicitly show the baseline differences between regions, so we added a violin plot that illustrate these large baseline differences. We also replaced the bar plots with distributional plots for the example psueudopopulation and added distributional plots showing across 1000 random pseudopopulations. (Note that Supplemental Figure 1 is now Figure 2 in the revised manuscript.) The figure now appears as follows:

**“Figure 3: Heterogenous changes in the sparsity of neural activity across regions.** *A) The Gini index is a measure of the sparsity of the distribution of spikes across a neuronal population. As more spikes are concentrated in a smaller number of neurons (light trace), the area under the curve (shaded region) increases, increasing the Gini index. B) The distribution of Gini indices across pseudotrials in one example pseudopopulation, across OFC (purple), VS (orange), and DS (green). Dots = mean across pseudotrials, bars = 95% CI. C) Distribution of Gini indices plotted separately for rule-free (red) and rule-based (blue) pseudotrials in OFC (left), VS (middle) and DS (right). Dots = mean across pseudotrials, bars = 95% CI. Asterisks = p < 0.02, two-sample t-test. D) Distribution of the mean Gini indices for rule-free and rule-based trials across 1000 pseudopopulations. Dots = mean across pseudopopulations, bars = 95% CI. Asterisks: p < 0.05, paired bootstrap test.”*

**Typos: L77 "requires"**, **L97 we?**, **L756 the?**

We have corrected these typos and thank the reviewer for altering us to them!

**Reviewer #2:**

**In this paper, Ebitz and colleagues present a study in monkeys learning stimulus-response mappings with reversals. A nice feature of the task is that three alternatives are provided for each choice dimensions, whereas many previous studies have focused on binary choices. Neuronal recordings are analyzed though little information is provided for motivating a focus on these particular regions. The paper overall is clearly written. I present below a few general comments, and then I develop more specific comments.**

We appreciate the reviewer’s kind words about the design of our our study and the clarity of our presentation. The reviewer raised a wide range of important points and we believe that addressing these has produced a substantially improved manuscript.

**General comments

The authors present evidence that they interpret as consistent with a "warping hypothesis". However too often, it is not explained or demonstrated why the findings invalidate or are inconsistent with the alternative "handoff hypothesis", which seems to me not enough to support the claims. Otherwise both hypotheses could be consistent with the findings. One exception is Fig 3C, but it is quite an aggregate metric, could the authors provide more of such contrasts? Or rewrite the conclusions appropriately?**

**In particular the main finding that rule-relevant dimensions under rule-based, if I have understood correctly, does not seem to me contradictory with a handoff hypothesis. It remains to be demonstrated whether the results would also be compatible with an alternative explanation, for instance attentional gating that the authors touch upon briefly in their discussion.**

**Because only three regions were recorded, the title and conclusions seems to me too affirmative and general. I appreciate the value of such recordings, and the practical constraints, but it remains possible that other areas would be compatible with an alternative handoff explanation, as has been proposed before (this discussion point could be further developed). Because the three areas show similar patterns on most results presented, it remains possible that other areas would show patterns consistent with a handoff hypothesis instead.**

This reviewer reiterates a point shared with Reviewer #1, that the automatic/deliberative “handoff hypothesis” was not as fully developed as the warping hypothesis. This reviewer also points out here and in later comments that we cannot definitively reject the handoff hypothesis, which could still be happening in regions we did not record from, for example. In response to comments from both reviewers, we decided to remove discussion of the handoff hypothesis and refocus the paper on the warping hypothesis that motivated the vast majority of our analyses and conclusions.

We have made many changes to the manuscript to address this concern, including a re-written introduction, the removal of hypotheses illustrated in Figure panel 3C, and the removal of all references to the handoff hypothesis from the text and figure legends. There changes did not substantially alter the interpretation of the data, but did increase the clarity of the manuscript.

The revised introduction reads as follows:

**“**We have a tremendous capacity to change how we perceive and respond to the world as our environment and goals change. One central part of this flexibility is our ability to learn rules: simple policies for guiding behavior that allow us to make decisions quickly, reasonably accurately, and with little subjective sense of effort (1–4). We know from many studies that rule identity is encoded in firing rate changes of neurons in specific brain regions, classically in the dorsolateral prefrontal cortex (dlPFC; (1–4), but also in regions implicated in decision-making, like the orbitofrontal cortex (OFC) and striatum (5–10). However, implementing a rule requires more than simply encoding its identity in the right structures and an important question remains unanswered: how do rules shape neural processing?

One possibility is that rules warp the way the world is represented in decision-making circuits to facilitate rule-relevant computations. Many decisions require us to solve very high-dimensional problems, where the correct response is some complex function of our past choices, reward history, and the various features of the options available to us in the moment (11). However, computation takes time and energy (12), especially when performed with metabolically expensive spikes (13, 14). Thus, in practice, we use various strategies to simplify decision-making (15–17). For example, we may execute a win-stay-lose shift strategy where we need only keep track of the last choice and its outcome, then compare the features of new options against the last choice according to the rule determined by the last outcome. There is no need calculate the value of each alternative or to represent information acquired further back into the past. Stimulus-response rules could simplify decision-making even more. To decide based on a well-learned stimulus-response rule, one need only determine whether or not an option satisfies the rule, which could be as simple as picking a berry that has the right color. This eliminates the need to represent or perform computations on any rule-irrelevant information and could thereby reduce the energetic and metabolic costs of decision-making (11, 15, 16, 18, 19).

Although stimulus-response rules could simplify decision-making in theory, it is not clear if the brain can take advantage of this opportunity. One influential hypothesis is that the brain implements rules and other executive functions as a kind of attentional gate that enhances the representation of relevant features of the world at the expense of irrelevant features (20). Eliminating the early sensory encoding of irrelevant information would certainly reduce the costs of decision-making. However, tests of this hypothesis in both prefrontal cortex (21, 22) and early sensory cortex (23, 24) have failed to find evidence of early feature selection. Information about irrelevant features of behaviorally relevant stimuli is still strongly encoded in these regions, even when it can interfere with task performance (4, 21, 22). Further, while feature-based attention can enhance the responses of sensory neurons that are tuned to prefer attended features (25–31), this does not mean that feature-based attention can selectively enhance a neuron’s response to one feature of a stimulus but not another. In fact, attending to one feature of a stimulus can enhance information about its other features, even when they are behaviorally irrelevant (23, 32–34). If we are trying to pick the ripest berry, can we really choose only based on color in order to take advantage of the computational efficiency that could be gained through ignoring shape and size?”

**How were these recording sites selected in the first place?**

These regions were selected by Dr. Brianna Sleezer for her dissertation project based on hypotheses she had about switching in reward circuitry (see her dissertation or Sleezer and Hayden, 2016; Sleezer et al., 2016; Sleezer et al., 2017). The hypotheses tested here were devised by Dr. Ebitz later, after the data were collected.

**Regarding the efficiency argument, while the authors present evidence that making choices under a rule require less resources (the experimenters observe less spikes) than on rule-free trials, what about the resources dedicated to the acquisition of the rule i.e. learning stimulus-response mappings in the first place? Could the authors explain why they do not take into account the neural resources to encode the rule per se that in turn will save resources when responding? Could the rule be learnt in the initial blocks or even training, and then subjects retrieve them when appropriate?**

This is an interesting idea. Unfortunately, we did not record while the animals were training on this task, so our data cannot speak to the energetic costs of acquiring a rule in the first place. We have added a line to the first paragraph of the discussion that acknowledges this limitation:

“Rules were well-learned before data collection began, so these results do not exclude the possibility that acquiring a rule has substantial metabolic demands, but they do resonate with the idea that we use rules, in part, because applying them reduces the energetic costs of decision-making.”

In the future, we do hope to collect the data needed to provide a satisfying answer to the question of how rules (and their metabolic demands) develop with training.

**As I caveat I should say that because a lot of references are made to previous papers in terms of experimental paradigm, computational model, behavioral analyses, I could not review these in detail; having not worked with pseudopopulations myself, I am not able to appreciate whether this is a relevant approach here, though it does seem appropriate and it is well introduced and justified indeed by the authors.**

We appreciate that reviewing previously published material in addition to the present manuscript would take an inordinate amount of time and agree that the paper would be best served by not leaving the reader with the sense that they have to read three papers to understand one. In several places in the ensuing comments, the reviewer highlighted ways in which we can make this paper more of a stand-alone document, and we have made these suggested changes. This includes adding detail of our HMM method, our approach to formal analysis of behavior in this task, and our methods for dimensionality reduction.

**Specific comments

Introduction

The introduction could be clearer regarding what is the question addressed: is it warping vs. handoff? Is it about testing the idea that warping is an efficient use of resources? It is unclear how this notion of efficiency is defined, and as compared to what.**

We apologize that our introduction was not as clear as it should have been. We have completely rewritten it (it was copied above in response to a previous comment). Part of the goal of our revision was to address this comment, clarifying 1) the central question we are addressing, 2) what we mean by efficiency and how it relates to the idea of representational warping. We hope that the reviewer finds that the revised introduction clarifies these issues.

**The last part of the introduction does not explain why it is critical to compare following a rule when it is the correct rule vs. when it is not the correct rule. Could this be conflated by amount of reward received (more reward received when you follow the correct rule)?**

We apologize for the confusion. We did not compare periods where following a rule is correct versus when it is not correct (which we agree is conflated with reward). It looks like our language in this section was not clear, so we have amended it as follows:

“Previous studies have typically examined the neural basis of rules by comparing periods in which one rule or another is imposed by the task (1–4, 7, 22, 36–38). These studies then define rule-based decisions as the correct decisions that occur once rules have been learned. However, here, we took a computational approach, which modeled rules as the latent stimulus-response policies underlying the monkeys’ decisions (39). This allowed us to the neural correlates of these latent rule states, as well as to contrast rule-based decisions with decisions that could not be explained by a simple stimulus-response rule.”

**Results

It remains possible that other computational models would also match the choice features very well. The model predicts rule-free trial after reversals, as expected when subjects are figuring out the new rule. It means that subjects' learning curves are very fast, with most their choices matching the true underlying state. Could the authors provide justification for their HMM approach, and explain why there is no model comparison?**

We have added more justification for our approach, including model comparison to the paper. It is important to note that the goal of the HMM is to label rule-based decisions in order to differentiate them from other types of decisions. Therefore, we compared the model against other approaches to doing exactly that. The HMM is agnostic to the computations underlying behavior by design, so it does not make much sense to compare the performance of the HMM at labeling trials with the performance of a generative model at explaining the computations underlying behavior. We describe the additional analyses in the text as follows:

“To infer what rule, if any, subjects were applying on each trial, we used a hidden Markov model (HMM; see **Methods**; **Figure 1C**). Previous rule-based sequential decision-making studies have operationally defined rule-based choices as the set of correct choices within each correct feature block. However, in this task, this approach would 1) include some unambiguously non-rule-based choices (i.e. correct responses made while guessing), and 2) exclude some unambiguously rule-based choices (i.e. perseverative rule-based decisions after the end of the block). We developed an HMM to resolves these issues, which ultimately produced trial labels that better explained variance in neural activity than these traditional approaches (**Supplemental Figure 1**).”

Supplemental Figure 1 now shows a direct comparison between the HMM and two more traditional approaches to separating rule-based decisions from other decisions.

***“Supplemental Figure 1: The HMM explains neural activity better than other approaches.*** *Related to Figure 1 and 2. Multiple approaches have been used to identify the rule-based decisions in tasks where the correct rule changes over blocks. The most common approach is to identify rule-based choices as the correct choices that occur within a block. Alternatively, and particularly in tasks where rule changes are uncued, rule-based choices may be identified as the correct choices that occur after some initial burn-in or learning period (i.e. after 5 trials). Here, we instead modeled rules as the latent states underlying decisions in an HMM, then used the HMM to infer which decisions were most likely to be rule-based. To determine whether this approach was appropriate, we used model comparison to ask whether the rule labels inferred from the HMM (dark gray) better explained variance in neural activity than rule labels derived from more traditional approaches (all correct choices within a block = light gray, all correct choices after a 5 trial burn-in period = middle gray). In model comparison, the model with the lowest AIC and BIC values is the preferred model. Within each region, AIC and BIC values were both lowest for the HMM, indicating that this approach explained the most variance in neural activity. The AIC and BIC weights for all the alternative approaches were less than 10^-32^, indicating very strong evidence that the HMM approach was the best.”*

Detail of this new analysis now appears in the methods:

***“Model comparison***. We used model comparison to determine whether the state labels inferred from the HMM better explained neural activity better than other approaches we could have used. Firing rates of each neuron *i* were modeled as Poisson distributed, with the mean rate dependent on whether or not each trial *t* was rule-based:

The logical “rule” was defined in one of three ways: 1) rule versus not rule as inferred from the HMM, 2) correct versus incorrect choices, 3) correct responses 5 or more trials after the block change versus other choices. We then calculated the log likelihood of the three models and, from that, their Akaike information criterion (AIC) and Bayesian information criterion (BIC) values (Matlab: aicbic.m). AIC and BIC provide measures of the relative likelihood of sets of models, but their units are arbitrary, so they are transformed into relative likelihoods (AIC or BIC weights) for quantitative comparison.”

**Could the authors further quantify the distribution of rule-free choices instead of giving only some percentages: they are a third of all trials. Out of that third, how many are after a reversal (5 first trials of a block) or in a stable phase (rest of the block, as defined by the authors)? Out of these, how many correspond to correct vs. incorrect trials? Statements like « very often » are not enough, it is important to provide quantitative findings for readers to get a better sense of the behavior.**

We have added the added quantification and analyses as follows:

“Rule-free choices occurred more often, but not exclusively, at block changes (**Figure 1D**). On average, across sessions, 45% of rule-free choices (± 21% STD, 95% CI across sessions: 12 to 81%) were within 5 trials of a block change, significantly greater than the expected frequency of 21% (± 4%, t(1,127) = 14.8, p < 0.0001, paired t-test). Conversely, 35% of rule-free choices were during stable periods (10 or more trials from a change point, ± 21% STD, 95% CI across sessions: 5 to 76%), significantly less than the expected frequency of 57% (± 8%, t(1,127) = -15.9, p < 0.0001, paired t-test). This suggests that rule-free choices increased in frequency when the subjects needed to explore in order to determine what rule to follow.”

The information about the accuracy of these choices appears in the next paragraph:

“For example, rule-free decisions were correct 49% of the time (± 6.9% STD), regardless of when they occurred in the block. That is, rule-free choices right after a block change were correct about half the time (47% ± 7% correct, within 5 trials of a block change), and so were rule-free choices during stable periods (52% ± 13% correct, 10+ trials after).”

**The statement that rule-free decisions by the model are actually « strategic, deliberative decisions » and not errors has to be demonstrated. Could the authors better explain why rule-free decisions being correct 49% of the time is inconsistent with a mixture of random choices and errors?**

Figure 1E-G, new Supplemental Figure 2, and 4 paragraphs of the results, including a section now entitled “Rule-free decisions maximize reward and/or information”, all make the point that these decisions were not errors, but rather were strategic and deliberative (in the sense that they maximized reward and/or information and appeared to do so via using information about previous choices and outcomes). Because errors would only be correct 3% of the time (the probability that an error of rule-maximization would correspond with an uncued block change and happen to target the correct feature), and random choices are only correct 33% of the time (1/3 chance of being correct by random selection), any possible mixture of errors and random choices could not be correct any more than 33% of the time (100% random, 0% errors) or less than 3% of the time (0% random, 100% errors). We have modified this section as follows to make this point more clearly (p 5):

“Rule-free choices were correct far more frequently than we would expect from errors (3% correct), random decisions (33% correct), or any combination of errors and random decisions (which would be bounded between 3% and 33% correct; **Figure 1E**). Errors would be correct approximately 3% of the time because this is the probability that an error of rule-following would coincide with a block change and target the new correct feature (significant difference: p <0.0001, t(1,127) = 77, one-sample t-test). Similarly, random decisions would only be correct 33% of the time (p < 0.0001, t(1,127) = 26.9, one-sample t-test; both effects survive correction for multiple comparisons). Thus, there is no way to produce choices that are correct 49% of the time through a simple combination of errors (3%) and random decisions (33%). This level of performance is impressive because the largest proportion of rule-free choices occur in the first few trials after block changes, when the animals could not know what the correct feature was. This implies that the monkeys were using an intelligent strategy—perhaps some form of hypothesis testing—to accelerate their ability to learn during rule-free choices.”

In the original manuscript, Figures 1E-G made the point that rule-free decisions are, on average, strategic and intelligent, and we have now added Supplementary Figure 2, which unpacks our reasoning in more detail. The relevant panels show, through formal analysis of the task (described in the Methods and pasted in response to a later comment below), what the optimal choices would look like (i.e. what choices would either maximize information or maximize reward), then illustrate how closely the monkeys follow this policy:

**Supplemental Figure 2: Rule-free choice patterns balance reward and information maximization.** A) The expected information gain (left) and expected reward probability (right) for choosing an option that differs in 0, 1, or 2 dimensions from the previous choice, given that the last trial was rewarded (cyan) or not rewarded (gray). The blue dotted lines shown here reflect the combination of these two functions, given the typical reward history on rule-free trials (i.e. a weighted average of the two functions, reflecting the fact that 52% of rule-free trials follow reward delivery). B) The choice patterns illustrated in Figure 1G, now plotted separately for trials following reward omission (left) and reward delivery (right). Conventions are the same as Figure 1G. Arrows reflect the choices that would maximize information (black arrows) or rewards (blue arrows), given that choice history, as illustrated in the previous panel.

In part because the task is complex and the stimuli are multidimensional, this policy is necessarily more complex than a win-stay lose-shift strategy (though we might also class win-stay-lose-shift as an intelligent strategy in the right circumstances). The relevant section of the results reads:

“The strategy used, on average, during rule-free trials tended to maximize information and/or reward. For example, after reward omission, choosing a novel option—one that differs in both color and shape from the last choice—maximizes both reward likelihood and information about the correct feature (**Figure 1H**; see **Methods** for details on how the information- and reward-maximizing choices were identified). Thus, the optimal choice after reward omission by either criteria is to chose a novel option. On the first trial after feedback that the correct feature has changed, subjects made this optimal choice 95% of the time (± 6.7% STD across sessions, sig. different than chance, p < 0.0001, t(1,127) = 50.6). Similarly, across all rule-free trials where the subjects were not rewarded on the last trial, regardless of when these occurred within the block, they made the same novel choice 94% of the time (± 6.8%; **Figure 1F**; p < 0.0001, t(1,127) = 46.7). Moreover, rule-free choices in general, regardless of whether they followed reward omission, were strongly biased towards the options that would maximize information, as calculated from the the actual pattern of choices and rewards that preceded these choices (probability of changing 1 dimension from the previous choice: 32.6% ± 4.6% STD, paired t-test for different than chance at 17.3% ± 2.5% STD, calculated based on how often this option was presented in each session: p < 0.0001, t(1,127) = 48.2; mean difference in probability: 34.5% more than chance, 95% CI = 14.6 to 15.2%; **Figure 1G, Supplemental Figure 2**; see Methods). Thus, rule-free choices used a strategy that integrated information about past rewards and choices to maximize information about what rule was best.”

**It is also not surprising that animals are above chance after a reversal of rule, because rewards indicate that the current rule is no longer valid via reward feedback, so they switch to a new rule. But the chance level then is not really 33% correct, because subjects are now choosing between the rest of the alternative rules i.e. hypothesis testing (all minus the rule they have just switched away from)?**

If the monkeys are excluding options that matched the previous rule, they are not making random decisions—they’re excluding options based on their knowledge of the task and their history of choices and outcomes. Though they may still choose randomly between equivalent options (i.e. between all the novel options), truly random decision-making would not exclude any option. (Also we should note that avoiding features consistent with the previous rule could hinder performance in this task since the stimuli are multidimensional and new combinations of features are shown on every trial). We agree that the strategy that the monkeys were using would perhaps be better described as a “hypothesis testing” strategy, where monkeys are using information about previous choices and outcomes to guide the choices that they make after block changes. We have amended this line to use the language suggested by the reviewer:

“This level of performance is impressive because the largest proportion of rule-free choices occur in the first few trials after block changes, when the animals could not know what the correct feature was. This implies that the monkeys were using an intelligent strategy—perhaps some form of hypothesis testing—to accelerate their ability to learn during rule-free choices.”

We also return to this point in the discussion:

“Many, but not all of the rule-free choices resembled a kind of exploratory hypothesis testing process, where a noisy win-stay lose-shift strategy was used to cycle through possible rules until the correct one was found (59–61).”

**Instead of a deliberative strategy, could it be a simple pavlovian avoidance of choice features that were relevant before towards new choice features? The fact that monkeys made that choice often is not enough evidence that they engage in a strategic, deliberative thinking.**

Our sense is that conditioned responses are typically slow to develop and even slower to extinguish, especially when rewards are omitted (as is the case here), rather than replaced with punishment. Thus, changes in behavior in response to one-trial-back feedback that we focus on here would be difficult to explain in terms of the literature on conditioned responses. However, it is possible that our animals “learned to learn”, meaning that through their training on this task, they learned to change their strategy quickly at change points. Our sense is that this kind of meta-learning would typically be thought of as more of a complex, cognitive process rather than the result of conditioning, however. Further, avoiding previously relevant features would interfere with performance in this task, because the stimuli are multidimensional and new combinations of features are shown on each trial. This means that previously relevant but devalued features must sometimes be selected in order to discover the new rule. Ultimately our argument is not that their decisions were smart because they avoided choice features that were previously relevant, it’s because they flexibly adjusted their behavior in response to single wins and losses to make the correct, information-maximizing choice. To clarify this point in the manuscript, we have added substantial detail of the monkeys’ strategy to the results section, which we detailed in response to the reviewer’s previous comments.

**Relatedly in Fig 1F, is the strategy different from a simple win stay lose shift?**

Choosing a novel option after a loss avoids both of the features of the previous choice, and thus could be consistent with a lose-shift strategy (provided the animals also tend not to chose these same novel options after a win, which we now show is true in Supplemental Figure 2B). However, as we wrote in response Reviewer #1, who made a similar point, 1) the evidence for the win-stay side of the equation is not so clear, in large part because 2) a win-stay lose-shift strategy is not sufficient to guide decision-making for multidimensional stimuli. For example, monkeys most frequently decide to switch one dimension, while staying in the other in this task. This is simultaneously a switch decision and a stay decision, so it’s hard to fit this choice into the taxonomy of win-stay-lose shift. However, we do like the idea that their behavior may reflect a win-stay-lose-shift strategy in hypothesis-testing space: that they test the hypothesis that blue is the correct feature, then pursue that strategy if they were rewarded, but not if they were not rewarded. We added a line to the discussion to raise this possibility:

“Many, but not all of the rule-free choices resembled a kind of exploratory hypothesis testing process, where a noisy win-stay lose-shift strategy was used to cycle through possible rules until the correct one was found (59–61).” **Beginning of page 5: it is unclear if the comparison bears on all trials or only on the first trial after a change.**

We have amended the language in the two paragraphs on page 5 to be more clear that we are referring to all rule-free choices, not to any subset of rule-free choices. The line “For example, rule free decisions were correct 49% of the time (± 6.9% STD)” has been amended to specify that this includes all rule-free decisions, “regardless of when they occurred in the block.” We also provide evidence that the timing of rule-free decisions with respect to block changes had little impact on their accuracy, which we referenced in response to a previous comment, but will paste again here: “[…] rule-free choices right after a block change were correct about half the time (47% ± 7% correct, within 5 trials of a block change), and so were rule-free choices during stable periods (52% ± 13% correct, 10+ trials after).” We also added the “regardless of when” clause to related line in the next paragraph “Similarly, across all rule-free trials where the subjects were not rewarded on the last trial, regardless of when these occurred within the block, they made the same novel choice 94% of the time (± 6.8%; Figure 1F; p < 0.0001, t(1,127) = 46.7).”

**I disagree with or have misunderstood the authors' reasoning that after an error, it is a strategic choice to change both color and shape features at the same time: because if the choice is then rewarding, it will be difficult to infer whether it is due to the shape or to the color (credit assignment problem). Instead, if the subject changes one feature at a time, it can test whether that feature is relevant (as the authors nicely show in the mutual information inset).**

It seems this section was not sufficiently clear from comments from both Reviewer 1 and 2. The inset in Figure 1G illustrates the information gained from changing 0, 1, or 2 choice dimensions during rule-free choices in general—regardless of whether they follow reward omission or reward delivery. However, the information gained following reward omission and delivery differs, as we now show in the relevant panels of new Supplemental Figure 2 (shown in response to a previous comment, but pasted here again to illustrate this new point).

**Supplemental Figure 2: Rule-free choice patterns balance reward and information maximization.** A) The expected information gain (left) and expected reward probability (right) for choosing an option that differs in 0, 1, or 2 dimensions from the previous choice, given that the last trial was rewarded (cyan) or not rewarded (gray). The blue dotted lines shown here reflect the combination of these two functions, given the typical reward history on rule-free trials (i.e. a weighted average of the two functions, reflecting the fact that 52% of rule-free trials follow reward delivery). B) The choice patterns illustrated in Figure 1G, now plotted separately for trials following reward omission (left) and reward delivery (right). Conventions are the same as Figure 1G. Arrows reflect the choices that would maximize information (black arrows) or rewards (blue arrows), given that choice history, as illustrated in the previous panel.

So, after a reward omission, the choice that would optimize either information or reward is the one that changes both features from the previous choice. As the reviewer notes, this is different from the choice that maximizes information after reward delivery – where changing only one feature of the last choice maximizes information, while changing zero features maximizes reward. Critically, the monkeys flexibly adjust their behavior in response to the last outcome so that they have a preference for making the information-maximizing choice either way. (Following losses, we cannot distinguish information maximization from reward maximization because both strategies would make the same choice, so we are generally agnostic in the manuscript about whether these choices are, on average, maximizing information, reward, or both.)

In addition to adding these figure panels, we have also added language and a new equation to the methods section that describes our approach to formal analysis of this task (“Identifying information-maximizing choices”). We hope this helps clarify that Figure 1G is a weighted average of the choice pattern following wins and losses (which is illustrated more fully in Supplemental Figure 2): “The reward-maximizing choice, conversely, is the one that maximizes the probability of reward. We calculated these values via simulating sequences of choices, then calculating belief states, belief state entropy, and estimated reward probabilities. In order to ensure that information would be computable, small amounts of noise, |Ν(0, 10^-4^)|, were added to all 0’s in the belief states before these were renormalized into valid probability distributions. Choices were then classified as differing in 0, 1, or 2 stimulus features from the last choice and the average information gain across these reward-history classes, weighted by their relative frequency during rule-free choices, is illustrated in the inset of **Figure 1G**. That is:

Where the p(r_t-1_=1) is simply the observed probability that the monkeys were rewarded on the trial immediately before a rule-free choice (i.e. 0.52).”

**Isn't it incorrect to label these choices as « optimal », because it depends on what is actually optimal: deciding as fast as possible? Number of rewards obtained?**

The reviewer makes an important point that any use of the term “optimal” should always be explicit about what function is being optimized. Here, we used the term to mean the optimal choice on a given trial, where that choice is the one that maximizes both the probability of reward and information about what feature is currently best (which, in turn, will maximize the number of rewards over longer time scales). This claim is based on formal analysis of the task, is a fairly standard definition of optimality, and is clearly and carefully described in the text of the revised manuscript:

“For example, after reward omission, choosing a novel option—one that differs in both color and shape from the last choice—maximizes both reward likelihood and information about the correct feature (**Figure 1H**; see **Methods** for details on how the information- and reward-maximizing choices were identified). Thus, the optimal choice after reward omission by either criteria is to chose a novel option. On the first trial after feedback that the correct feature has changed, subjects made this optimal choice 95% of the time (± 6.7% STD across sessions, sig. different than chance, p < 0.0001, t(1,127) = 50.6).”

New Supplemental Figure 2 illustrates this point explicitly, which we have already shown/described in response to previous comments.

It is important to note that we use the term “optimal” only in this very circumscribed context where we have the formal analyses needed to support this claim. We did not consider response time effects because these trials are very long (> 3 seconds) and include plenty of time for deliberation (subjects can make decisions long before they can report them). Making decisions 20-30 ms faster would not substantially improve the rate of reward here. Of course, we are quite sympathetic to the idea that optimal decision-making in a broader view will also minimize its costs, not only in terms of time, but also in terms of their energetic and metabolic demands. (Indeed, this perspective motivated much of the paper!) However, we don’t have the formal analyses needed to make any claims about optimality outside of this one circumscribed instance.

**p. 5: « Rule-free trials in general were strongly biased towards… » again no quantitative information or statistical test is provided to support this statement.**

We have added a statistical test to support this statement to the results:

“Moreover, rule-free choices in general, regardless of whether they followed reward omission, were strongly biased towards the options that would maximize information, as calculated from the the actual pattern of choices and rewards that preceded these choices (probability of changing 1 dimension from the previous choice: 32.6% ± 4.6% STD, paired t-test for different than chance at 17.3% ± 2.5% STD, calculated based on how often this option was presented in each session: p < 0.0001, t(1,127) = 48.2; mean difference in probability: 34.5% more than chance, 95% CI = 14.6 to 15.2%; **Figure 1G, Supplemental Figure 2**; see Methods).”

**It would be useful to look at the information on Fig 1G not on average but for trials following a block change. It is not demonstrated how such choices maximize reward too, not only information gain.**

Panel A of new Supplemental Figure 2 (pasted again here for clarity) shows the reward and information gain following reward delivery and omission separately. Although panel B focuses on how the previous reward changes the pattern of decision-making, we saw strikingly similar effects for the first trial after a block change is indicated by reward omission (this was illustrated in Figure 1F in the original manuscript and now in more detail in Supplemental Figure 2C, which was included to address this comment). This is perhaps not surprising given that subjects could only infer where they were in a block though their experienced reward history.

“**Supplemental Figure 2: Rule-free choice patterns balance reward and information maximization.** A) *The expected information gain (left) and expected reward probability (right) for choosing an option that differs in 0, 1, or 2 dimensions from the previous choice, given that the last trial was rewarded (cyan) or not rewarded (gray). The blue dotted lines shown here reflect the combination of these two functions, given the typical reward history on rule-free trials (i.e. a weighted average of the two functions, reflecting the fact that 52% of rule-free trials follow reward delivery). B) The choice patterns illustrated in Figure 1G, now plotted separately for trials following reward omission (left) and reward delivery (right). Conventions are the same as Figure 1G. Arrows reflect the choices that would maximize information (black arrows) or rewards (blue arrows), given that choice history, as illustrated in the previous panel. C) Same as B, for all choices after the first omitted reward after a block change.”*

**Could the authors show whether the distinction rule-based/rule-free does not simply reduce to a « after reversal »/« plateau phase », just in terms of correctness, following the rule of the current block vs. not?**

The distinction between rule-based and rule-free does not reduce to after-reversal versus plateau phase because subjects could fail to learn a rule or spontaneously stop using a rule during the plateau phase (rule-free during plateau) or they could either perseverate on an old rule or discover the new rule unusually quickly after a block change (rule-based after a block change). Further, model comparison results (shown in response to a previous comment) suggest that contrasting correct trials during stable periods with other trials is a poor substitute for our computational approach (Supplemental Figure 1).

**Could the authors present the results in Fig 3D separately for automatic and deliberative choices? Because most choices in the block will be automatic ones, they are the not discriminative choices for arbitrating between the two hypotheses.**

This question appears to be due to a misunderstanding of the hypotheses illustrated in Figure 3C, which we have since removed from the manuscript. Figure 3D plots the results associated with the “automatic/deliberative” regions illustrated in 3C, but it showed no evidence of a distinction between OFC, VS (deliberative), and DS (automatic). To avoid confusing future readers and, given the changes to the framing of the manuscript (i.e. we have removed all discussion of the “handoff hypothesis”), we have removed the hypothesis plot in Figure 3C and replaced it with the neuronal tuning results requested in the minor comments from the reviewer.

**Could another explanation for the discrepancy with fMRI findings is that the authors have recorded from a limited number of areas (understandably) as compared to whole brain measurements?**

The handoff hypothesis was about fMRI results in the specific regions we were recording from (OFC, VS, and DS), so it seems unlikely that the discrepancy is due to neural activity in other regions. In the interest of streamlining a dense manuscript, we have removed reference to the handoff hypothesis, but we do still mention the discrepancy between our results and previous BOLD-imaging work in the results. Our sparsity results (Figure 2) provide one explanation for the discrepancy that we find particularly compelling, but we take the reviewer’s point that its important to present alternative explanations as well. Therefore, we have added the following concluding sentence to this section of the results:

“This suggests that differences between **Figure 2** and results of related work in fMRI (43–45) may be due to differences in what is measured by the BOLD signal versus single unit electrophysiology, though we cannot rule out alternative explanations, like differences between tasks, species, data analysis methods, or limitations in our spatial sampling within OFC, VS, and DS.”

**In Fig 4B does the conclusion boils down to the fact that after a reversal, more uncertainty so RSA more scattered, then more compact for rule-based when contingencies have been learnt?**

This seems unlikely given the differences between regions. Representational space is more compact in OFC during rule-free choices. However, it is expanded in VS and unchanged in DS (see the second column of Figure 4C, which quantifies this directly). Because we see similar changes in feature-matching vs non-feature matching representational similarity in all three areas, and not just in OFC, a change in the compactness of the representation is probably not driving the effects across all three regions. To the question of uncertainty—unfortunately these results cannot really speak to that because this task is poorly suited to measure uncertainty (rewards provide perfect information) and because rule-free choices are not exclusively occurring when uncertainty is high. This is an interesting idea for future projects, but unfortunately one that we cannot test with this dataset.

**Could the authors discuss the lack of OFC modulation in rule-free choices: is OFC selective for rule-based as a cognitive map of task space (Wilson et al. 2014)?**

This question has two parts. For the first part, it’s not clear to us what result this question refers to. In all critical analyses, we saw a significant result for the OFC that mirrored the other two regions. For the second part, the answer is yes. Specifically, we believe that Wilson et al.’s perspective provides a useful one for understanding OFC, and, indeed our results are consistent with that view. That said, testing his theory was neither the goal of the present study nor a problem that the present study can provides a definitive answer to. As such, we believe the safest, most conservative route is not to make any major claims about the cognitive map model. We did add a citation to this paper in the discussion, noting that “nearly 90% of neurons in OFC encoded the identity of the current rule, which is a striking proportion. This resonates with modern views that OFC is responsible for tracking the current state of dynamic environments (60) and causal results that suggest that lesioning OFC disrupts learning that requires state inference (61).”

**In terms of writing, it would be useful if the authors further unpack their reasoning from the results to the implications or conclusions and claims that they derive. There was often a bit of a gap or a large step between the finding and a general conclusion or claim that followed.**

We have gone through the manuscript with an eye towards unpacking our reasoning in each section and clarifying how the results follow from the conclusions. This included a number of changes in the introduction, results, and discussion. Each section of the results now starts with a description of what question this analysis will address and concludes with a sentence or two explaining the significance of the result, using the same language that we will return to in the discussion. These changes are too numerous to quote succinctly here, but we have marked all changes in the revised manuscript.

**Discussion

Could the authors engage more with the alternative explanations that they propose for their findings, in particular an attentional gain, and what would be the role of the other areas mentioned (dACC, DLPFC)?**

We added detail to this section of the results, specifically discussing how the results could reflect a change in neuronal gain coming from dACC or a categorization signal coming from dlPFC:

“A change in sensory encoding is not the only way to produce changes in how choice information is represented in neural populations (22, 35). The effects we report here could also be due to other selective mechanisms, including changes in the intrinsic dynamics of populations of decision-making neurons (22, 35), an adjustment in neuronal gain coming from dorsal anterior cingulate (72, 90), or amplification through recurrent interactions between these regions and/or through the thalamus (91). One particularly compelling possibility is the idea that they reflect a category bias that OFC, VS, and DS may share with or inhereit from dorsolateral prefrontal cortex (20). Prefrontal neurons can flexibly categorize objects with respect to arbitrary rules, meaning that neural responses to sample stimuli are more discriminable according to rule-relevant category boundaries than irrelevant boundaries (92, 93). Here, we find broadly similar effects in OFC, VS, and DS in a very different type of task, suggesting that these representational changes 1) are more broadly distributed than was previously known, and 2) occur spontaneously during rule-based decision-making. Of course, categorization is not necessary for good decision-making, even in this task, and we are more than capable of representing every dimension of these decision-making problems (22), so it is interesting to consider the possibility that these signals reflect the outcome of some kind of categorization process.”

We also now include the following new text in the discussion:

The results we have collected are necessarily limited in scope to OFC, VS, and DS, but they do raise the question of how other regions would behave. This question is especially salient for two key dorsal executive regions, dorsal anterior cingulate cortex and dorsolateral prefrontal cortex. These regions are often treated as the locus of the central executive, suggesting that they would be likely to serve a regulatory role over the regions in our study (20, 64–67). However, an alternative view is that these regions are part of a larger network specialized for converting sensory information to actions (68–72). In that view, we would predict that these regions would show largely similar functions to OFC, VS, and DS. Indeed, a recent study by our lab suggests that dACC may also show rule-specific dimensional warping (73).

**How do these results relate to the identified dissociation between lateral PFC, medial PFC, OFC and striatum identified in humans and non-humans for rule-based and rule-free trials (e.g. Collins & Franck, 2014; Walton et al., 2010)?**

It’s not clear to us what the reviewer is asking about. First, no previous study has contrasted rule-based to rule-free decisions, as the math to do so was developed by us for this study. Neither paper uses those terms, so it’s hard to tell what the reviewer is referencing. Moreover, Walton’s paper considers OFC not striatum, and Collins’ paper is limited to striatum. Our results also have very little to say about lPFC or mPFC because we have no recordings from these regions. More broadly, we understand that there are identified differences between OFC and striatum, and find these to be credible. Our results focus on a domain in which they are not particularly different, but these results – and the corresponding interpretation we offer – depend critically on novel analyses that were not available to past scholars, so our work is, in our view, complementary, not contradictory.

**Could the authors address rule acquisition (cf. general comment), at least comment on or speculate how this transition may occur? If space permits the discussion should also comment on the behavior and the model, especially in relation to extensive previous work on set acquisition and exploration.**

The reviewer raises a very important topic, one that is superficially similar to the one our paper covers. However, on reflection, we believe the topic is actually quite different - as different as decision-making and reward-learning. Unfortunately, we don't believe that our data shed much light on the important topic of rule acquisition, simply because we did not record during the acquisition period. The discussion does briefly allude to this as a limitation of the present paper: Rules were well-learned before data collection began, so these results do not exclude the possibility that acquiring a rule has substantial metabolic demands**.** However, we do not feel comfortable speculating further.

**Minor comments

Even if the HMM has been described elsewhere, it would be useful to have a list or a graph depiction of the Markov process and relevant variables**

We agree with the reviewer’s point that this manuscript should provide sufficient detail to understand the HMM without having to make reference to previous publications. We address this concern in multiple ways. First, we have added an unrolled illustration of the HMM (which illustrates all dependencies) to our depiction of the transition matrix in Figure 1C, copied here:

We also amended the caption for this revised figure panel to read:

*“C) Features of the hidden Markov model used to identify rule states. Left) An unrolled illustration of the model. Choices, illustrated as colored stars, are modeled as emissions from a hidden process that is in one of various latent states at each time point (s_t_). Right) The transition paths between latent states in the model. Rule states only emit choices that match the state’s feature, while any choice can occur from the rule-free state.”*

Finally, we have also amended the methods section to provide additional detail of and intuition for the HMM:

“***Hidden Markov model (HMM):*** A hidden Markov model was used to infer the latent goal state underlying each choice. This model allowed us ask whether, for example, each blue-star choice was made because 1) it was a star, 2) it was blue, or 3) the monkey was searching for the correct feature or performing some other non-rule-based computation. We have previously used this method to identify latent goal states in other tasks (63) and described how this model was developed for this task (36).

In the HMM framework, choices (x) are “emissions” that are generated by an unobserved decision process that is in a latent, hidden state (z). Each hidden state has some observation model, which dictates the probability of choosing each option when the process that state. For example, the blue-rule state had an observation model where the probability of choosing the blue option was 1, but the probability of choosing a non-blue option was 0. Formally:

Conversely, because we do not know what monkeys would choose in a rule-free state, we default to the maximum entropy distribution. Thus, the observation model for any of the N=3 choice options (n) during rule-free states is:

Inverting the emissions model allows us to make inferences about the state the animal was in at the time when they made the choice. For example, a blue-star choice can only be due to a blue rule, a star rule, or a rule-free choice, but not to a magenta rule. We can refine this inference further by taking the temporal structure of the hidden states into account.

Because the model is Markovian, the probability that monkeys will be in any state z at time t depends only on the immediately preceding state (Figure 1C), that is, the model assumes that states are conditionally independent of all previous states and choices, given observation of the most recent state:

The temporal dependencies between states are represented by a stochastic matrix known as a “transition matrix”, whose entries are the 1-time-step probability of transitioning from each state to every other state, including itself. We reasoned that monkeys could not divine the new rule following a change point and instead had to explore to discover it, so direct transitions between different rule states were fixed at 0 in the model. For the same reason, the monkeys were also assumed to start in the rule-free state on the first trial each day. The structure of the transition matrix is illustrated in Figure 1C.

Transition parameters were tied across the six rule states, meaning that the model ultimately had only 2 free parameters: the probability of staying in the rule-free state and the probability of staying in any of the six rule-based states. Fitting these two parameters allows the model to account for the natural temporal dynamics of the behavior. For example, rule-based states had a strong probability of transitioning to themselves, so they tended to be occupied for many trials in a row, while the rule-free states tended to be briefer. We can use these insights to refine our inference about the hidden state for our blue-rule choice. If a blue star is chosen now, and the last choice was a blue circle (due either do a blue rule, a circle rule, or a rule-free state), then the most probable explanation, given these temporal dynamics, is that we are currently in a blue rule state. The next most probable explanations are that we just observed 2 rule-free states in a row, or a transition from a star-rule to a rule-free state, but if the next choice is a blue option or two trials back was also a blue option, then these alternative explanations become less and less likely.

These two parameters were fit to each individual session via expectation-maximization using the Baum Welch algorithm (96, 97). This algorithm finds a (possibly local) maxima of the complete-data likelihood, which is related to the joint probability of the hidden state sequence and the sequence of observed choices. The algorithm was reinitialized with random seeds 100 times, and the model that maximized the observed (incomplete) data log likelihood was ultimately taken as the best for each session. We then used the Viterbi algorithm to identify the most probable a posteriori latent state underlying each choice (97), given the entire sequence of choices and the parameters of the fitted model.”

**Could the authors indicate, out of all neurons recorded regardless of task properties, what is the proportion that present activity related to any task property (for each area)?**

We have added figure panel 3A that illustrates the tuning for the three task properties we focused on in this paper: rule use, rule identify (blue rules, star rules), and choice identity. We didn’t perform any pre-selection, so this plot does include all of the recorded neurons.

The legend reads as follows: “*A) The proportion of single neurons in each region that were tuned for rule use (differentiated rule-based from rule-free choices), rule identity (the color or shape rule underlying rule-based choices), and choice identity (the color and shape of the chosen option). Error bars = binomial standard error, dotted line = proportion expected by chance.”*

The original manuscript reported the proportions tuned for choice: Neurons in all three areas encoded the visual features of the upcoming choice (“choice identity”; **Figure 3A-C;** one-way ANOVAs, predicting choice identity from the firing rate of each neuron; VS: n = 52/103, proportion = 0.50, p <0.0001; DS: n = 85/204; proportion = 0.41, p <0.0001; OFC: n = 52/115, proportion = 0.45, p < 0.0001; one-sided binomial test for difference from expected false positive rate of 0.05).

We now also report the proportion tuned for rule-use: A large proportion of individual cells differentiated between rule-based and rule-free trials in each region (**Figure 3A**; one-way ANOVAs within each neuron; VS: n = 45/103, proportion = 0.44, p <0.0001; DS: n = 72/204; proportion = 0.35, p <0.0001; OFC: n = 49/115, proportion = 0.43, p < 0.0001; one-sided binomial test for difference from expected false positive rate of 0.05).

And the proportion tuned for rule identity: Indeed, just over 80% of of single neurons encoded rule identity (**Figure 3A**; one-way ANOVAs within each neuron, excluding rule-free trials; VS: n = 82/103, proportion = 0.80, p <0.0001; DS: n = 157/204; proportion = 0.77, p <0.0001; OFC: n = 102/115, proportion = 0.89, p < 0.0001; one-sided binomial test for difference from expected false positive rate of 0.05).

**Fig 2a, could the authors add coordinates info regarding recording sites**

We have added a detailed account of the location of all the recording sites to the manuscript. Because this required a substantial amount of text, we added the text to the methods section, and added a note to the figure caption for Figure 2A to direct the reader to the methods section. The revised caption now reads: “Coronal slices illustrating the approximate center of recording sites in the OFC, VS, and DS. See Methods for detail of the spatial extent of recording sites.”

The revised methods section now reads: “OFC recordings were made at gray matter sites between 0 and 9 mm above the ventral surface, lateral to the medial orbital sulcus, and between 29 and 36 mm rostral of the coronal plane containing interaural zero. VS recordings were made at gray matter sites between 0 and 8 mm from the ventral surface of striatum, between 0 and 9 mm lateral to the midline plane, and between 28 and 21 mm rostral of the coronal plane containing interaural zero. DS recordings were made at gray matter sites more than 8 mm from the ventral surface of striatum, between 0 and 9 mm lateral to the midline plane, and between 28 and 21 mm rostral of the coronal plane containing interaural zero. Recording locations and the locations of significant anatomical boundaries (the ventral surface of striatum, the medial orbital sulcus) were verified through a combination of Brainsight (MR) guidance and listening for characteristic sounds of white and gray matter while lowering the electrodes, which in all cases matched the boundaries predicted by the Brainsight system.”

**Figure legends: could the authors keep « bars » for bars and indicate « error bars » or « shaded areas » where appropriate**

We have corrected these errors in the figure legends.

**« Was due to » p. 13 no correlation/causation is proved**

We apologize for this misstatement. We have amended this sentence to read: “Thus, there was an increase in the representational similarity of choices that differed solely along rule-irrelevant dimensions, consistent with the predictions of our overarching hypothesis.”

**A « transmission matrix » should be a transition matrix (HMM section)**

Thank you for catching this error. We have corrected this to section to use the correct term. (all uses of “transmission” corrected to “transition”)

**Fig 4B / 5A color scale as legend should be symmetric otherwise the color indicating 0 is misleading**

We have replaced these graphs with versions where the color scale is symmetric around 0.

**Mutual information: clarify methods section: « Two firing rate bins per neuron were used because maximized the number of observations per condition »**

This passage lacked necessary detail. The revised manuscript provides the detail of our rationale: Two firing rate bins per neuron were used for two reasons (though the results were similar with more firing rate bins). First, it allowed us to include all the cells. There were a small number of very sparsely firing neurons whose spike counts could not be quantile binned into more than two bins (i.e. they did not fire during this epoch approximately half the time, but, when they did, fired between 1-3 spikes). Second, it maximized the accuracy of our probability calculations. To perform these analyses, we had to estimate a minimum of 36 probabilities (i.e. combinations of 2 choice types, 9 choice identities, and at least 2 firing rate bins) from a finite sample of trials. As the number of bins increases, the number of observations per bin goes down and it becomes increasingly difficult to determine whether a bin is empty because its probability is truly 0 or because we simply did not observe it in our finite sample.

**"While this idea makes intuitive sense" is not a very scientific statement**

This sentence does not appear in the revised introduction.

**Fig 3B, please clarify in the legend what are the grey shades representing**

We have added the following to the caption “Shading represents z-scored firing rate and the color bar is shared across plots.”

**Methods section, category tuning: shouldn't the noise be epsilon(i,k) for trial k? Please clarify.**

Yes, it should be epsilon(i,k). Thank you for catching our error in these two equations (methods section entitled “Category and identity tuning in single neurons”).

**Reviewer #3: Main comments:**

**This is a nice manuscript with some interesting and unique observations.  In particular, the contrast between rule-based and rule-free modes is interesting and noteworthy.  The differences in between the striatum and OFC are also quite noteworthy.   In general, a very nice set of results that will be of interest to anyone interested in decision-making.

I wonder, however, how much some of the interpretations of the results are a little oversold.  The authors conclude that rule-adherence "warps" neural activity.  This is certainly the case but is it a new observation of a re-packaging of old observations?  It seems to me that the warping can be explained by already observed phenomenon, specifically feature-based attention and dimensionality reduction (categorization).  The authors allude to prior work on these phenomena but I, for one, could not get my head around how "warping" is different.  I am open to being convinced otherwise but the authors need to address this explicitly.**

We appreciate the reviewer’s kind words about the quality of the manuscript and the breadth of researchers who will be interested in the paper! The reviewer also raises one major concern here: that idea these results might be due to already-known phenomena, like feature-based attention in and/or categorization. We believe our results are a novel contribution to the literature, rather than a repackaging of old results, for two reasons.

**First,** it was not a foregone conclusion that rule-based decision-making would evoke patterns that look like feature based attention and/or categorization. Indeed, that is one major result here! Our task doesn’t require subjects to use feature-based attention—the stimuli are presented sequentially, which we know minimizes attentional selection, and there are no demands for sustained attentional selection in the task. Further, even the final saccadic choice could be made just based on the remembered location of the stimulus that was chosen during the sequential phase. Our task also doesn’t ask monkeys to make categorical judgments—there is no need to assign options to categories to chose between them and we know from previous work on multi-dimensional decision-making (and the rule-free trials here) that we are more than capable of representing two different attributes of a choice simultaneously.

**Second,** we are recording in OFC, VS, and DS. All three are regions in which we still don’t know how feature-based attention and/or categorization works. It would be very interesting to know if these regions parallel patterns seen in extrastriate cortex or dorsal prefrontal cortex–it would suggest that basic principles of feature-based attention, for example, are not confined to classic attention regions, but instead appear even in regions that are highly abstracted from the sensory signal. Unfortunately, we cannot currently say how these regions respond in classic feature-based attention and/or categorization tasks. We also cannot say if this task evokes similar changes in activity in regions more classically linked to attention and categorization, but these are two exciting future directions for this work.

There is also a third, more subtle, reason why the results shown in the original version of the manuscript are not a re-packaging of feature-based attention: feature-based attention is the enhanced sensory representation of one stimulus at the expense of another, but the analyses in the original manuscript focused on how choice information is represented in the brain, not sensory information. We absolutely agree with the idea that changes in choice information could be due to the effects of feature-based attention on sensory encoding, but this has to be demonstrated. The revised manuscript begins to do exactly that.

Because we are excited about the idea that feature-based attention could be part of the mechanism by which we make rule-based decisions, we have added a new section to the results which asks whether there is any evidence for a change in feature-based attention in this task. In short, we do find modest, but significant changes how neurons are tuned for stimulus features during the sequential presentation phase: tuning sharpens for rule-relevant features compared to rule-irrelevant features. These effects are not large enough to explain away changes in how choices are represented, but they could be nonlinearly amplified in decision-making circuits, for example. This new section of the manuscript is pasted below:

**Changes in single neuron tuning functions suggest a combination of early and late selection**

Together, the results suggest that rule-based decision-making expands the representation of choice dimensions that are rule-relevant, while compressing dimensions that are rule-irrelevant. Superficially, this appears to support the influential idea that executive processes may recruit selective visual attention to enhance the processing of rule-relevant stimulus dimensions at the expense of irrelevant dimensions. This is because early gating of the sensory signal would presumably propagate forward, affecting the way that the world is represented in every region that inherits sensory input. However, these are changes in choice representations, not stimulus representations, and, as such, could be due solely to late changes in the decision-making process itself (22, 35) rather than early changes in visual attention. Attention is the selection of one stimulus for enhanced processing at the expense of another (26, 57, 58). Therefore, to determine whether adhering to a rule also modulated visual attention, we next asked whether rules altered the selectivity of the neural response to stimuli.

Here, three options were presented sequentially in advance of the decision. These options were composed of random combinations of color and shape. This meant that we could measure the neural response to every stimulus color and shape on every trial, during the three 400 ms epochs when each option was presented alone on the screen. Asynchronous presentation is not typically ideal for measuring the effects of visual attention, because attentional enhancements are largest when multiple stimuli are presented in competition (59). However, our hypothesis was that the different *features* of the same stimuli would be in competition for attentional selection. Based on previous studies of feature-based attention using unidimensional stimuli (26, 28), we reasoned that feature-based attention would enhance neuronal selectivity for selected, rule-relevant stimulus features at the expense of selectivity unselected, rule-irrelevant features. Indeed, this is what we saw.

A small, but insignificant number of individual cells were tuned for either the color or shape of the option on the screen (two-way ANOVA with main effects of color and shape fit to each neuron, 10.2% of neurons, 43/442, were significant for either color or shape at p < 0.05, not significantly greater than the expected false positive rate of 9.75%). The rule-relevance of the feature dimension modulated this minimal tuning (**Figure 8A**; generalized linear model, see **Methods**; significant interaction between feature preference and feature relevance: beta = 0.01, p < 0.05; significant main effect of feature tuning: beta = 0.03, p < 0.0001; nonsignificant main effect of feature relevance: beta = -0.02, p = 0.07). Within each feature dimension, rule-relevance increased the difference in firing rate between the response to the best feature (i.e. the stimulus shape or color associated with the highest firing rate across all epochs) and the response to the worst feature (shape rule: color tuning = 0.061 z-scored firing rate units ± 0.092 STD, shape tuning = 0.087 ± 0.107, mean pair-wise difference = 0.026, paired t-test: p < 0.0002, t(1,421) = 3.92, 95% CI = [0.013, 0.039]; color rule: color tuning = 0.078 ± 0.150 STD, shape tuning = 0.062 ± 0.174, mean pair-wise difference = 0.017, paired t-test: p < 0.05, t(1,421) = 2.42, 95% CI = [0.0004, 0.033]; both effects survive correction for multiple comparisons). No difference between color and shape tuning was observed during rule-free trials (color tuning = 0.080 ± 0.096, shape tuning = 0.077 ± 0.115; mean pair-wise difference = 0.003, p > 0.6). Changes in stimulus tuning were quite modest in magnitude and were not observed within individual regions, when treated in isolation. However, early changes in sensory encoding did exist and thus could have contributed to changes in choice representations.

In order to directly compare the magnitude of these small, early changes in sensory encoding with the changes in choice representations explored previously, we next characterized the magnitude of the effects of rules on choice representations in these same epochs. We performed the same procedure as before, but now looked at how the neurons were tuned to features of the ultimate choice, instead of to the features of the stimuli on the screen. As expected, rule relevance modulated neural tuning to features of the choice (**Figure 8B**; significant interaction between feature preference and feature relevance: beta = 0.21, p < 0.0001; significant main effect of feature tuning: beta = 0.027, p < 0.0001; significant main effect of feature relevance: beta = 0.11, p < 0.0001). Again, within each feature dimension, rule-relevance increased the difference in firing rate between the response to the best feature (highest firing rate) and the response to the worst (lowest firing rate; shape rule: color tuning = 0.059 z-scored firing rate units ± 0.132 STD, shape tuning = 0.296 ± 0.320, mean pair-wise difference = 0.24, paired t-test: p < 0.0001, t(1,421) = 14.66, 95% CI = [0.20, 0.27]; color rule: color tuning = 0.245 ± 0.324 STD, shape tuning = 0.047 ± 0.154, mean pair-wise difference = 0.196, paired t-test: p < 0.0001, t(1,421) = 10.20, 95% CI = [0.16, 0.23]; both effects survive correction for multiple comparisons). Thus, the effects of rule-relevance on choice feature tuning were an order of magnitude larger than the effect of rule-relevance on stimulus feature tuning, within in the same restricted epochs. Together, these results reinforce the central result that rule-based decision-making expands the representation of choice dimensions that are rule-relevant, while compressing dimensions that are rule-irrelevant. However, they also suggest that early changes in selectivity for stimulus features could contribute to these effects.

**Figure 8. Effects of rules on neuronal tuning for stimulus features and choice features.** *A) Neuronal tuning for the color (left) and shape (right) of presented stimuli during the three 400 ms epochs when these stimuli were on the screen, plotted separately for color rule, shape rule, and rule free trials. “Best” and “worst” refer to the the stimulus features associated respectively with the highest or lowest firing rate within neurons. B) Same as A, but sorted by choice features, rather than stimulus features. Note the change in the y-axis needed to illustrate this substantially larger effect. Error bars = standard errors across 422 neurons. Note that these may be smaller than the symbols.*

We have also added a discussion of the relationship between these results and the feature-based attention/categorization literature to the discussion, so that future readers can get a sense of how we see these results fitting into that literature:

The idea that cognitive processes warp how the brain represents information has support in both influential theories of prefrontal function (20) and modern views at the intersection of executive function and selective visual attention (53, 58, 83–85). Our results strongly support the idea of representational warping: showing that rules not only gate which stimuli are represented in the neural code (86), but also *how* different stimuli are represented. This result is particularly significant because several previous single neuron studies did not report evidence that task demands can gate or warp the representation of low-level sensory features in the brain ((4, 21–24), though compare with fMRI results (87, 88)). Future work is necessary to determine whether the critical difference between our study and previous single unit studies is one of task design, analysis method, or the fact that this study targeted limbic regions commonly implicated in decision-making, rather than prefrontal or extrastriate regions linked to controlling or executing visual attention (21–24). Certainly, one straightforward way to produce broadly distributed representational changes would be through a change in visual attention, either at the level of the extrastriate cortex (26–28) or in the prefrontal regions that direct featural attention (89). Early feature selection could, in theory, alter the way that stimulus features are represented within every region that receives ascending visual information. We do report some evidence in favor of this idea here. However, like many attentional effects, early feature selection was modest and some kind of nonlinear amplifying process would still be needed to transform modest biases in stimulus representations into substantial biases in choice representations. Further, similar effects have not been reported in regions more clearly implicated in visual attention (4, 21–24) and it is still not entirely clear that feature-based attention *can* prioritize one feature of a stimulus at the expense of another (27–29, 32–34).

A change in sensory encoding is not the only way to produce changes in how choice information is represented in neural populations (22, 35). The effects we report here could also be due to other selective mechanisms, including changes in the intrinsic dynamics of populations of decision-making neurons (22, 35), an adjustment in neuronal gain coming from dorsal anterior cingulate (72, 90), or amplification through recurrent interactions between these regions and/or through the thalamus (91). One particularly compelling possibility is the idea that they reflect a category bias that OFC, VS, and DS may share with or inhereit from dorsolateral prefrontal cortex (20). Prefrontal neurons can flexibly categorize objects with respect to arbitrary rules, meaning that neural responses to sample stimuli are more discriminable according to rule-relevant category boundaries than irrelevant boundaries (92, 93). Here, we find broadly similar effects in OFC, VS, and DS in a very different type of task, suggesting that these representational changes 1) are more broadly distributed than was previously known, and 2) occur spontaneously during rule-based decision-making. Of course, categorization is not necessary for good decision-making, even in this task, and we are more than capable of representing every dimension of these decision-making problems (22), so it is interesting to consider the possibility that these signals reflect the outcome of some kind of categorization process.

**Mind you, this does not take away from a number of unique and important new observations.  The observation that the rule-free state is not just random guessing but is instead used maximize information gathering is nice.  The fact that this rule-free state vs the rule-based state engages the OFC and striatum differently is an important observation.  But the idea that there is attentional selection during rule-based behavior is not all that new, at least as disseminated here.  I guess the bottom-line is that the authors need to draw a sharper contrast and make more explicit how these observations differ from prior work showing feature-based attention and categorization (dimensionality reduction) shown previously.** 

We appreciate the reviewer’s careful reading of the manuscript and efforts to highlight its various other novel contributions. We hope we have successfully clarified our view that the representational warping is not a foregone conclusion of existing literature on feature-based attention and categorization, though we do see the intersection of these topics is an exciting direction for our research in the future.

**Minor comments:
1. There are multiple t-tests used for comparisons.  It is more elegant and proper to use one test like an ANOVA (or other non-parametric stats, if warranted) and then post-hoc contrasts to identify where the differences lie.  That reduces Type 1 errors. As far as I can tell, the t-tests were not corrected for multiple comparisons, which exacerbates the problem.**

We agree with the reviewer and have added more precautions against Type 1 errors. These did not change our results.

In the original manuscript, we took some precautions like directly asking whether the rate of positive tests is higher than the expected false positive rate of that family of tests (with a one-sided binomial test, standard practice in the field) and confirming that there are systematic biases in the sign of the effect across independent tests (because performing tests on noise would yield no such bias). In the revised manuscript, we have also added corrections for multiple comparisons in numerous places and ANOVAs where appropriate. The methods now describe our global approach to correcting Type 1 error rates as follows:

To correct for multiple comparisons when calculating the proportion of neurons tuned for different task variables, we used a one-sided binomial test against the expected false positive rate. Elsewhere, we used the Benjamini-Hochberg procedure (94). For independent tests, this is guaranteed to produce a Type I error rate at or below the family-wise rate, set at 0.05 by convention.

For tests where we could not use ANOVAs because we were examining independent sets of dependent variables, we have confirmed that the p-values of related sets of tests survive a Benjamini-Hochberg correction for multiple comparisons. The phrase “all effects survive correction for multiple comparisons” has been added to the text wherever we performed this correction. The correction was applied to the 1) behavioral results, 2) all mutual information analyses, 3) sparsity analyses, 4) all the comparisons of the rule-based/rule-free representational similarity results across regions, 5) all the comparisons of the color-rule/shape-rule representational similarity results across regions, and 6) the choice-predictive subspace projection results. Most of the p-values in the manuscript were already on the order of 10^-3 or less and no significant effect became not significant after correction for multiple comparisons.

The reviewer also points out that in places, we jumped directly to pairwise t-tests without performing the ANOVAs needed to justify these post-hoc tests. We have revised the manuscript to add these ANOVAs where appropriate. These new analyses further confirmed the results reported in the original manuscript. For the representational similarity analyses, we have added description of the new model to the methods section:

To determine whether there was significant structure in the representational similarity matrices shown in **Figure 5** and **6**, we fit ANOVAs to the upper triangular of the matrices (excluding the diagonal, which was all ones by definition). The ANOVA for **Figure 5** (rule-free versus rule-based) included three terms: 1) within versus between choice types, 2) within rule versus within search (nested in the within versus between predictor), and 3) sharing a feature versus not sharing a feature. The ANOVA for **Figure 6** (color rule versus shape rule) included a similar set of three predictors: 1) within versus between choice types, 2) within color rule versus within shape rule (nested in the within versus between predictor), and 3) sharing a color versus sharing a shape. In this second model, we omitted cells that matched both shape and color but not rule type because the predictor matrix would not have been full rank since we had to remove the diagonal of ones. All predictors were modeled as categorical variables and the models included all possible interaction terms.

The relevant sections of the results are, for Figure 5:

There was a representational shift between rule-based and rule-free decisions such that similar choices were represented by more similar patterns of activity during rule-based decisions (ANOVA, main effect of within/between choice type: F(1,147) = 138.9, p < 0.0001; main effect of rule-based/rule-free choice type: F(1,147) = 5.0, p < 0.03; main effect of matching features: F(1,147) = 23.3, p < 0.0001; interaction between within/between and matching features: F(1,147) = 7.9, p < 0.006; interaction between rule-based/rule-free and matching features: F(1,147) = 32.0, p < 0.0001).

For Figure 6:

Combining across OFC, VS, and DS, we saw significant change in the organization of neural representations across rule types (ANOVA, main effect of within/between rule type: F(1,135) = 131.1, p < 0.0001; main effect of rule type: F(1,135) = 0.12, p > 0.7; main effect of matching features: F(1,135) = 43.6, p < 0.0001; interaction between within/between and matching features: F(1,135) = 44.4, p < 0.0001; interaction between rule-type and matching features: F(1,135) = 172.4, p < 0.0001).

The other place where an ANOVA would have been good to include to motivate a series of post-hoc t-tests is in the choice-predictive subspace projections in Figure 6. The relevant section of the methods describes this ANOVA:

To determine whether rules had any effect on where neural activity lay in the choice predictive subspace, we fit an ANOVA with two terms: whether the decision was rule-based or rule-free decision, and then, nested within that, whether the axis was relevant to the rule, irrelevant to the rule, or if it was rule-free and we had no knowledge either way.

And the results are as follows:

The magnitude of choice-coding-dimension projections depended on the subjects’ goal: whether they chose the stimulus because the category was relevant to the rule, irrelevant to the rule, or the result of rule-free decision-making (Figure 7D; two-way ANOVA, main effects of rule-free versus rule-based: F(1, 1077) = 28.1, p < 0.0001, main, nested effect of whether the feature was relevant to the rule: F(1, 1077) = 229.4, p < 0.0001).

**2. The authors state that reducing neural activity tends to reduce information.  This is not really true.  First of all, neurons with tuning curves tend to convey more information when they are not at peak excitability.  And there are examples in the literature of neurons firing more sparsely, yet being more finely tuned after learning.**

The reviewer makes an important point that our discussion of the relationship between firing rate and mutual information was overly simplistic. We had focused on one specific problem: the fact that reducing mean firing rate across conditions would bring neural activity closer to the low/no-spiking noise floor, which would reduce mutual information. However, we did not discuss the fact that sharpening tuning functions could produce both an increase in mutual information and a decrease in mean firing rate, when firing rate is averaged over multiple conditions. Our view, which we believe is in agreement with the reviewer’s point, is that sharpened tuning functions are indeed driving both the increase in information and decrease in neural activity in this data set and we have amended our discussion of this result in several places to make this point clear.

The results section has been rewritten to introduce the analyses as follows:

“Spikes are energetically costly (13, 14), so a distributed decrease in neural activity during rule-based decisions could reflect decrease in the energetic costs of rule-based decisions, compared to decisions that require navigating more complex decision-spaces. Alternatively, the decrease in spike rates could suggest that OFC, VS, and DS are just disengaged during rule-based decisions—perhaps they fire fewer spikes because they are simply encoding less information, for example. Ultimately, a more efficient code requires both 1) that fewer spikes be expended (as we have seen), and 2) that information not be lost, despite this decrease in firing rate.”

Also, the line “First, mutual information typically *increases* with mean firing rate” has been revised to read “First, in the absence of any changes in tuning functions, we would expect mutual information to *increase* with mean firing rate because increasing spike counts moves activity away from the noise floor (48, 49).” To the end of this paragraph, we have also added a brief sentence highlighting the reviewer’s point that this probably suggests a change in the way information is encoded in these neurons: “This may suggest that using a rule changes the way that neurons are tuned for choice in these regions.”

We return to this point in the discussion (p 18): The original line “Further, although we know that reducing neural activity tends to reduce information (49), choice information was actually increased during rule-based choices.” was revised to read: “Further, although firing rates were reduced during rule-based choices, information about the features of the chosen option was increased—suggesting rule-based choices were more metabolically efficient than non-rule-based choices.”
