# Supplementary material for: Rules warp feature encoding in decision-making circuits
Source: PLoS Biol. 2020 Nov 30;18(11):e3000951. doi: 10.1371/journal.pbio.3000951 (PMC7728226; doi:10.1371/journal.pbio.3000951)
Supplement: Attachment — Submitted filename: round2_R2R_ruleEfficiencyPlosBio.docx [file pbio.3000951.s009.docx]

**Academic Editor:**

**1. I agree with reviewer 1. The term "rule-free" is somewhere between confusing and nonsensical. I also find their rebuttal disingenuous: that WSLS is not an adequate description of the "rule-free" trials because the monkey strategy is more complex. The very existence of what they call "rule-free" trials shows that the monkeys' strategy is more complex than following the 6 ground truth rules, but it doesn't invalidate them!**

**They need to drop the "rule-free" terminology in favour of a more neutral term. What is special about those trials is that they can't be explained by the model the authors have chosen to use. That's all. In other words, they are most likely a grab-bag of things that the monkey is doing that includes some idiosyncratic randomness, structured exploration, and heuristics like WSLS. This needs to be acknowledged. I suggest using the term "idiosyncratic" or "residual" or some such epithet which does not make strong claims about what is going on there. The authors are painting themselves into a corner with the term "rule free" because it obliged them to argue that, despite the fact that the monkeys are adopting a behavioural strategy that is quite effective, it's somehow not reaonably described as a "rule", which seems extremely odd.**

We gather from the editor’s and Dr. Wilson’s comments that the term “rule-free” does not accomplish our goal of avoiding strong claims about this class of choices, even with the changes we made in the last round of revision. Therefore, we have changed this language to refer to “residual state/residual choices” throughout the manuscript.

We appreciate the effort that the editor and Dr. Wilson put into identifying terms that might be clearer than “rule-free”. After much discussion, our sense was that the editor’s second suggestion (“residual”) best described what these choices were (and were not) without making strong claims about what was going on during these choices. To address this comment, we have replaced all uses of the term “rule-free choices” with “residual choices” throughout the manuscript, equations, figures, and figure legends (or “rule-free decision-making” with “residual-state decision-making”). We are convinced this change improves the readability and clarity of the paper.

**2. Reviewer 2 originally pointed out that the the authors don't make a strong link between data and interpretation. When pushed to justify this, they simply removed one of the candidate explanations. I think a better way to deal with this is to honestly acknowledge any potential mismatch between data and theory. The reviewers are going to back this paper if the authors are straightforward, honest, and up-front about what their data do and do not say. So I would strongly suggest they orient the reader to clear, falsifiable predictions that either account would make for either hypothesis, and simply explain whether the data do (or do not) meet these. This would give the reader the opportunity to weigh up the evidence without excessive dressing up by the authors.**

The editor suggests that we add the handoff hypothesis back into the manuscript and we have done so. Because the editor and Reviewer 2 both seemed to find our decision to remove this hypothesis off-putting, we wanted to briefly explain our thinking from the last revision before we detail the changes in this revision. The hypothesis was an important part of our original motivation for this study (i.e. it informed our choice of regions and a few important analyses, particularly early on), but comments from Dr. Wilson and Reviewer 2 convinced us that 1) that other researchers might not agree with the interpretation of the literature that led us to come up with this hypothesis originally, 2) that the hypothesis added unnecessary complexity to the introduction, and 3) that readers might find any tests of this hypothesis unconvincing because we did not record from other regions that might play different roles in sensorimotor rule-guided decision-making. We were swayed by this feedback, so we took Dr. Wilson’s advice and removed explicit reference to a “handoff hypothesis” from the manuscript. (We should make clear that an abbreviated discussion of this hypothesis did remain in several places [e.g.. in our description of the sparsity results in Figure 3 and in the discussion], though we did not use the explicit “handoff hypothesis” terminology.) We hope that clarifying these points makes it clear that our intention in the last revision was not to misrepresent our work or work process, but instead to present that work in a way that minimized confusion and incorporated the critical feedback we received from the reviewers.

The changes we made seemed to improve the manuscript in Dr. Wilson’s view, but Reviewer 2 lamented the loss of this hypothesis as an explicit alternative and the editor agreed with Reviewer 2’s view. Therefore, we have revised the manuscript to follow the hybrid approach suggested by Reviewer 2 that includes this hypothesis as an explicit alternative, but limits mention of it in the results and discussion. We like this approach because it avoids the unnecessary complexity/confusion in the introduction (which we hope will satisfy Dr. Wilson), while preserving the logical justification for the regions we recorded from and the analyses we performed (which we hope will satisfy Reviewer 2 and the editor).

We now introduce the handoff-hypothesis in the results as follows:

“We recorded responses of individual neurons in OFC (n = 115 cells), VS (n = 103), and DS (n = 204; recording sites in **Fig 2A**). These regions were chosen because each is implicated in a different aspect of rule-guided decision-making. Dorsal striatum (DS) is implicated in rapid, stimulus-bound, automatic decisions (43–45), like the decisions that occur after a rule is well learned. Conversely, ventral striatum (VS) and the orbitofrontal cortex (OFC), are implicated in more flexible, deliberative decision-making (46–50), like the decisions that help us discover rewards or learn new rules in changing environments. Focusing on these regions allowed us to consider an alternative hypothesis about the neurobiological basis of rule-based decision-making: the idea that that rule-based decision-making involves a functional handoff from automatic decision-making structures, like DS, to more flexible decision-making structures, like OFC and/or VS (21–24). We will refer to this idea as the “handoff hypothesis” to differentiate it from the idea that rules are implemented via warping the distributed representation of choice information across regions.”

We then go on to describe what predictions the handoff hypothesis would make for the results that are relevant to this hypothesis. For example, we now introduce Figure 2 by stating:

“If rule-based decisions are more metabolically and computationally efficient than other choices, fewer spikes should be required to generate them across the distributed network of decision-making regions. However, the alternative, handoff hypothesis might predict a different pattern—like heterogeneous shifts in the concentration of neural activity across structures.”

In introducing Figure 3, we write:

“The fact that we observed similar firing rate effects in all three regions differs from what we may have expected from fMRI studies. fMRI studies (47, 49, 51) tend to report the kinds of complex shifts in activity between regions that we would expect from the handoff hypothesis: a dissociation between VS and OFC on one hand (where the BOLD signal is strongest during flexible decision-making and learning) and DS on the other (where the BOLD signal is strongest during rapid, stimulus-bound, automatic decisions, like the rule-based decisions here). This apparent discrepancy could reflect the fact that the BOLD signal may not scale with the rate of spikes, but instead with other population measures, such as how densely or sparsely activity is distributed across a population of neurons (52).”

We conclude this section by stating how the sparsity and firing rate effects in Figure 2 and 3 speak to the handoff hypothesis:

“Together, the firing rate and sparsity effects imply that fMRI evidence that supports a handoff in control may be due to inter-structure heterogeneity in how rules affect the density of spikes across neuronal populations, rather than heterogeneity in how rules affect net neural activity. Instead, across a distributed network of decision-making regions, we found that fewer spikes were needed to generate rule-based decisions.”

Figure 4 is, in our opinion, the strongest piece of evidence related to the handoff hypothesis in the paper. We explain why as follows:

“Spikes are energetically costly (13, 14), so a distributed decrease in neural activity during rule-based decisions could imply a distributed decrease in the energetic costs of rule-based decisions. This is precisely what we would expect if the brain takes advantage of the opportunity rules present to streamline decision-making. However, a more efficient code requires both 1) that fewer spikes be expended (as we have seen), and 2) that information not be lost, despite this decrease in firing rate. Alternatively, a decrease in spike rates could suggest that a structure is disengaged—perhaps fewer spikes are fired during rule-based decisions because less information is encoded in that structure. Further, if the handoff hypothesis is correct, the distributed decrease in net activity across OFC, VS, and DS could be due to different effects within each structure. Specifically, during rule-based decisions, net activity may go down in OFC and VS because the regions are disengaged (in which case choice information should also go down), whereas net activity may only go down in DS because there is less irrelevant information coming in from OFC and VS (in which case choice information in DS should go up). To differentiate between these possibilities, we next asked whether neurons in these regions encoded choice information and whether the energetic (spiking) cost of encoding this information was altered during rule-based decisions.”

This section concludes by stating:

“Because spikes are metabolically costly (13, 14), the fact that more information was conveyed per spike in every region we tested suggests that rule-based decisions may be more energetically efficient than the other decisions. Fewer spikes were wasted on computations that were irrelevant to the ultimate choice. This result is broadly consistent with the idea that rules can streamline decision-making, perhaps via distributed changes in the way that neurons are tuned for or representing choice information. We will look for these changes next. However, this result is not consistent with the predictions of the handoff hypothesis—where we would have expected an increase in information per spike in DS (because this region would be driving rule-based decisions), but a decrease in information per spike in VS and OFC (because these regions would have handed control off to DS).”

The remaining analyses test the additional, more stringent predictions of the warping hypotheses (and are essentially orthogonal to the handoff hypothesis), so we do not invoke this hypothesis again until the discussion. Here, we have added explicit reference to the “handoff hypothesis” back into a discussion of how these effects relate to the question of whether rules have similar, distributed effects across regions, or distinct effects within regions that would support the idea of a handoff in control:

“This means that we did not observe evidence for a handoff in control during rule-based decisions: there was no shift in the locus of choice-predictive information from VS and OFC (structures implicated in flexible, deliberative decision-making (28–32)) to DS (a structure implicated in automatized decision-making (25–27)). Although our results are not consistent with a functional handoff between these specific structures, we cannot rule out the possibility that there may be a functional handoff in other regions or the possibility that there was a shift in how the regions were communicating with each other. Further, we did observe several striking differences between regions, consistent with the results of previous studies that focused on the differences between these regions (47, 49, 51)...”

**Two comments of my own which seem substantive and were not raised at review, but which would set my mind at rest:**

**3. The authors see overall lower spike rates in the rule-based condition (when the monkey receives more positive outcomes) in three regions known to code for expected value . Is this a possible explanation?**

This is a critical concern – the idea that differences in expected value between rule-based and residual choices could have driven differences in spiking in these regions. However, we found that they did not. The manuscript explains the relevant analyses as follows:

“The decrease in activity during rule-based decisions was not due to differences in reward expectation between the two conditions because reward-dependent changes in firing rate were both smaller and in the opposite direction in all three regions (no reward - reward, VS: -0.28 ± 0.09, DS: -0.09 ± 0.07, OFC: -0.06 ± 0.07). Thus, if anything, we would be underestimating the extent to which adhering to a rule decreases spiking activity in these regions because rule-based choices were more likely to be rewarded than residual choices.”

**4. Can the authors confirm that the RSA results were done in cross-validation and avoid temporal confounds (neural signals for "rule-free" and "rule-based" are more similar within than between that designation because rule-free trials happen closer in time to other rule-free trials etc).**

The editor raises two important points, which we will address in turn. First, the editor points out that it is critical to know if the RSA results are robust to out-of-sample analysis. The original RSA results were not done in cross-validation, but we have corrected this in the revised manuscript, adding new Supplementary Figure 4. This shows that these results are indeed robust when the RSA is done by measuring distances between population representations in independent subsets of the data. These results closely resemble the other, non-cross-validated results in the paper (c.f. Figure 5, Supplementary Figure 3):

**Supplemental Figure 4: Cross-validated changes in choice representations across rule-based and residual-state decision-making*.*** *Related to Figure 5B and C. A) Distances between choice representations in non-overlapping subsets of trials. Within each cell, trials were partitioned at random into two equal subsets, with the constraint that each trial type was represented at least once in each subset. Then the two subsets were used to construct two independent psudopopulations. We then performed the same analysis as Figure 5B, but calculated distances between choice representations as the distance between the representation in one set and the representation in the other set. After in Figure 5B, this panel illustrates one example partition/pseudopopulation pair. B) The same analysis as Supplemental Figure 3, but distances are calculated from non-overlapping subsets of the data, as in panel A. Data is combined across regions. Dots = mean across pseudopopulations, error bars = 95% CI, asterisks = significant contrasts, all p < 0.0001. Specific contrasts, from left to right. Choice representations were closer to themselves within choice type than between choice types (mean difference = 0.72, 95% CI = [0.40, 1.02], p < 0.0001, one-sided bootstrap test). Mean representational difference was smaller during rule-based than residual choices (mean difference = 1.52, 95% CI = [1.11, 1.92], p < 0.0001). Residual choices that shared a feature were not closer together than choices that did not (mean difference = 0.01, 95% CI = [-0.31, 0.33], p = 0.48). However, rule-based choices that shared a feature were more similar than rule-based choices that did not share a feature (mean difference = 0.97, 95% CI = [0.68, 1.27]).*

The editor’s second point is that the temporal distribution of residual trials (was “rule-free”) and rule-based trials could complicate the interpretation of the RSA results. This is another critical point. For the results shown in Figure 5, pseudopopulations were constructed without regard to the specific rule, so rule-based choices that shared a feature were no more or less clustered together in time than residual choices that shared a feature. However, for the results shown in Figure 6, where pseudopopulations are based on color- or shape-rules, we have realized that the temporal structure does complicate the interpretation. This Figure shows that choices that share a feature and a rule domain (i.e. color or shape) are closer together in representational space than choices that share a feature, but not a rule domain. However, the former choices are also clustered together *in time*, whereas the later choices are not. To make the limitations of the interpretability of this result clear, we have added a substantial caution to the relevant paragraph of the results:

“Thus, there was an increase in the representational similarity of choices that differed solely along rule-irrelevant dimensions, consistent with the predictions of our overarching hypothesis. However, other interpretations are possible. For example, choices that share both a rule domain and a feature tend to be more tightly clustered in time than choices that share a feature, but not a rule domain. Temporal autocorrelations could also drive these similarity effects. Further, choices that share both a rule domain and a feature necessarily also share a specific rule—blue is the only color rule that would lead to blue-star, -circle, or -triangle choices. Any abstract encoding of a blue rule would also drive the representation of these choices to appear more similar.”

The next paragraph goes on to describe how similar effects were observed in all three regions individually, and we have added a similar caution to the end of this paragraph:

“though, again, other interpretations are possible, such effects due to the temporal structure of the task or abstract encoding of the current rule.”

**Reviewer #1, Charlie R E Wilson**

**The authors have considered my comments and those of the other reviewers seriously, and I think they have made significant strides in clarifying and improving the manuscript. I also find their interpretation more measured than previously, the figures improved and the extra supplementary stuff helpful. Their alteration of the theoretical context of the paper (in particular in response to reviewer 2 who made the point much better than I did) is appropriate and improves the paper. I still think there is much to like in this work, and that the manuscript merits publication.**

**I have one outstanding point to make. Perhaps the authors will find this pernickety as it is a point of language precision, but in re-reading the much-improved manuscript this phrase bothers me even more: we do not agree on the use of the term "rule-free".**

**Now, the manuscript is much clearer on what the authors mean by this phrase, and the authors have taken the time to write a thoughtful explanation of their arrival at this term. I also agree with their argument for the rejection of the term "exploratory" which I think we will probably agree is over-used, particularly in the context of monkey behaviour. It is much clearer that this collection of trials is being performed using adaptive rules or strategies, and that it is a heterogenous set of choices. The reasons to class these trials by a description of exclusion are clear. These are all vital improvements. But I'm afraid that the phrase "rule-free" is still misleading. We could presumably have a very interesting back-and-forth on this for the next few months, but to be brief I think there are two points that render the term *at best* ambiguous: 1. Many readers will indeed interpret "rule-free" to mean "random". 2. There are valid arguments that one should consider"performance rules" (like WSLS) to be rules, even if the authors wish to consider them strategies. The authors make clear several times that their full meaning here is "sensorimotor rule-free". We can all agree on this, so why not use this term throughout (perhaps "SM rule free"?) in order to be, as the authors note, crystal clear.**

We take this point, which was echoed by the editor. Our reasoning is given in detail above (see our response to the editor’s first point), but to restate briefly here, we take the point that the phrase “rule-free” remains a misleading distraction. Therefore, we now use the term “residual” (so “residual choices” or “residual-state decision-making”), which we hope emphasizes the fine point Dr. Wilson is making here: that saying a decision cannot be explained by a simple sensorimotor rule is not the same as saying that this decision was made without following a rule.

**I have comments about the authors' response to my comments on WSLS. On reflection I do very much take their point that it is really the SM rule-based trials that are the focus of the neurophysiological analyses of this study. I therefore agree that the brief comments on WSLS that they have added now suffice to cover the issue. I'm including these comments anyway for completeness.**

**The authors say "Because choices were multidimensional and the monkeys used a more complex strategy, a simple WSLS strategy isn't sufficient to explain our data" - this is pretty much an empirical claim for which the authors could provide the relevant analysis - I think there are versions of WSLS that can account for this behaviour across dimensions, particularly focussing on the lose-shift as the authors point out. The nature of the WSLS response depends what the animals have learned to shift *on*, and I think this is what the authors are getting at by their reference to "higher order" WSLS. This is *particularly* relevant in the context of the long and complicated testing history of these animals, something I think we all need to think more about in this field.**

Yes! This is exactly the point we were trying to make in the discussion--that the behavior resembles a win-stay lose-shift in hypotheses/rule space, rather than in action space. We have amended this section as follows to echo the reviewer’s language to try to be more clear on this point:

“This behavior resembled a classic win-stay/lose-shift in the sense that animals avoided repeating features after losses, but tended to repeat features after wins. However, because the stimuli were multidimensional and the most common response was to stay in one feature dimension while shifting in the other, a win-stay/lose-shift policy would have to be implemented at the level of hypotheses about what rule was correct, rather than at the level of stimulus responses to be successful in this task (79–81).”

**Reviewer #2**

**In their revision, Ebitz and colleagues have satisfactorily addressed most of my comments, and have made substantial efforts to clarify their paper. I have one remaining main comment, corresponding to my first original comment:**

**"The authors present evidence that they interpret as consistent with a "warping hypothesis". However too often, it is not explained or demonstrated why the findings are inconsistent with an alternative "handoff hypothesis", which seems to me not enough to support the claims. Otherwise both hypotheses could be consistent with the findings. In particular the main finding that rule-relevant dimensions under rule-based does not seem to me contradictory with a handoff hypothesis. It remains to be demonstrated whether the results would also be compatible with an alternative explanation."**

**It is too bad that the authors have made the decision to remove entirely the alternative "hand-off" hypothesis. This has left me wondering if these are actually post hoc explanations, not prior hypotheses to arbitrate between. Since the study hypotheses were not preregistered, the authors should show where the evidence is not only compatible with the warping hypothesis, but also incompatible with other (at least, the hand-off) hypotheses. In the current version, the authors have simply discarded alternative hypotheses, and only evidence supporting the authors' hypothesis is presented, which could be compatible with other hypotheses too. I think that even without fully going back to the previous version around these hypotheses, it is possible to include these pieces of evidence where appropriate in the Results section, and some discussion about it.**

We appreciate the reviewer’s point that the paper would be stronger with an explicit discussion of the alternative hypothesis that drove this work. We explained our reasons for removing in our response to the editor (above), but briefly--we removed explicit reference to this hypothesis at Dr. Wilson (Reviewer #1’s) suggestion because we thought this might make the paper more intelligible, but some discussion of this alternative viewpoint remained in the sections of the results and discussions where it was most relevant. However, we like the suggestion the reviewer makes here of a hybrid approach--where we introduce this hypothesis as an explicit alternative, giving it the space and detail it deserves, but confine discussion of it to the results and discussion so as to minimize the confusion/distraction from the main point of the paper.

Our revisions provide explication of the hypothesis, relevant citations, and context. There are also extensive revisions to the results that explain how the results support or challenge each hypothesis and a more developed discussion of the handoff hypothesis in the discussion. These revisions are detailed above in our response to the Academic editor, who makes the same point as the reviewer.

**And two remaining small comments:**

**- The authors have now included details about the anatomical landmarks for selecting their recording sites, but we are still told little about their motivation for focusing on these three regions, apart that they are broadly involved in decision-making (… like many other regions):**

**"We know from many studies that rule identity is encoded in firing rate changes of neurons in specific brain regions, classically in the dorsolateral prefrontal cortex, but also in regions implicated in decision-making, like the orbitofrontal cortex (OFC) and striatum." (introduction)**

**The paper would benefit from further justification here, especially as other (likely critical) areas are mentioned in their discussion.**

We have addressed this question in two ways. First, in the introduction, we explain our interest in the role of reward-associated regions in cognitive processes as follows:

“We have long been interested in the role of structures linked to reward in cognitive processes, and motivated by a belief that the distinction between motivational and cognitive processes is overly simplistic. For this reason, we were especially interested in looking at how rule-based decision-making is implemented in core reward regions, like OFC and VS, and in regions implicated in both reward processing and motor control/learning, like DS.”

Within the “core” reward regions, our specific choice of OFC, VS, and DS was motivated by the handoff hypothesis, so removing reference to this hypothesis likely made it harder to understand our choice of regions in the last revision. In adding this hypothesis back in, we were able to more clearly explain the rationale for our choice of structures when we introduce this hypothesis in the results:

“We recorded responses of individual neurons in OFC (n = 115 cells), VS (n = 103), and DS (n = 204; recording sites in **Fig 2A**). These regions were chosen because each is implicated in a different aspect of rule-guided decision-making. Dorsal striatum (DS) is implicated in rapid, stimulus-bound, automatic decisions (43–45), like the decisions that occur after a rule is well learned. Conversely, ventral striatum (VS) and the orbitofrontal cortex (OFC), are implicated in more flexible, deliberative decision-making (46–50), like the decisions that help us discover rewards or learn new rules in changing environments. Focusing on these regions allowed us to consider an alternative hypothesis about the neurobiological basis of rule-based decision-making: the idea that that rule-based decision-making involves a functional handoff from automatic decision-making structures, like DS, to more flexible decision-making structures, like OFC and/or VS (21–24). We will refer to this idea as the “handoff hypothesis” to differentiate it from the idea that rules are implemented via warping the representation of choice information.”

**- The authors often resort to very general terms like "intelligent", "clever" or "strategic", that can mean many things, and they do not specifically define what they mean here. For instance a WSLS can be strategic in certain contexts, as acknowledged by the authors. I recommend sticking to specific, precise language eg. « functions of the color, shape, and outcome of previous choices versus rule-based choices. ».**

We have revised the manuscript to remove terms like “intelligent”, “clever” and “strategic” wherever we found them. Following the reviewer’s suggestion, we now stick to specific and neutral language. For example:

Original text: “This implies that the monkeys were using an intelligent strategy—perhaps some form of hypothesis testing—to accelerate their ability to learn during rule-free choices.”

Revised text: “This implies that the monkeys were doing something more complex than random decision-making (like sequential hypothesis testing or avoiding previously unrewarded features), which accelerated their learning during residual choices.”

Original text: “The strategy used, on average, during residual choices tended to maximize information and/or reward.”

Revised text: “Residual choices tended, on average, to maximize information and/or reward.”

Original text: “In fact, the residual decisions identified by the HMM were often strategic, deliberative decisions that maximized reward and/or information about what rule was best.”

Revised text: “In fact, the residual decisions identified by the HMM often maximized reward and/or information about what rule was best.”
